# Supplementary material for: TIGIT can inhibit T cell activation via ligation-induced nanoclusters, independent of CD226 co-stimulation
Source: Nat Commun. 2023 Aug 18;14:5016. doi: 10.1038/s41467-023-40755-3 (PMC10439114; doi:10.1038/s41467-023-40755-3)
Supplement: Supplementary file 1 — Supplementary Information [file 41467_2023_40755_MOESM1_ESM.pdf]

Supplementary Information File

TIGIT can inhibit T cell activation via ligation-induced nanoclusters, independent of CD226 co-stimulation

Authors

Jonathan D. Worboys[1], Katherine N. Vowell[2], Roseanna K. Hare[1], Ashley R. Ambrose[1], Margherita Bertuzzi[3], Michael A. Conner[2], Florence P. Patel[2], William H. Zammit[1], Judit Gali-Moya[1,4], Khodor S. Hazime[1,4], Katherine L. Jones[1], Camille Rey[1], Stipan Jonjic[5], Tihana Lenac Rovis[5], Gillian M. Tannahill[6], Gabriela Dos Santos Cruz De Matos[6], Jeremy D. Waight[2] and Daniel M. Davis[1,4]

Affiliations

[1]Lydia Becker Institute of Immunology and Inflammation, Faculty of Biology, Medicine and Health, Manchester Academic Health Science Centre, University of Manchester, Manchester, UK
[2]GlaxoSmithKline, Collegeville, USA
[3]Manchester Fungal Infection Group, Faculty of Biology, Medicine and Health, Manchester Academic Health Science Centre, University of Manchester, Manchester, UK
[4]Department of Life Sciences, Sir Alexander Fleming Building, Imperial College London, South Kensington, London, UK
[5]Center for Proteomics, Faculty of Medicine, University of Rijeka, Rijeka, Croatia
[6]GlaxoSmithKline, Stevenage, UK

Corresponding author email

d.davis@imperial.ac.uk

Supplementary Figures

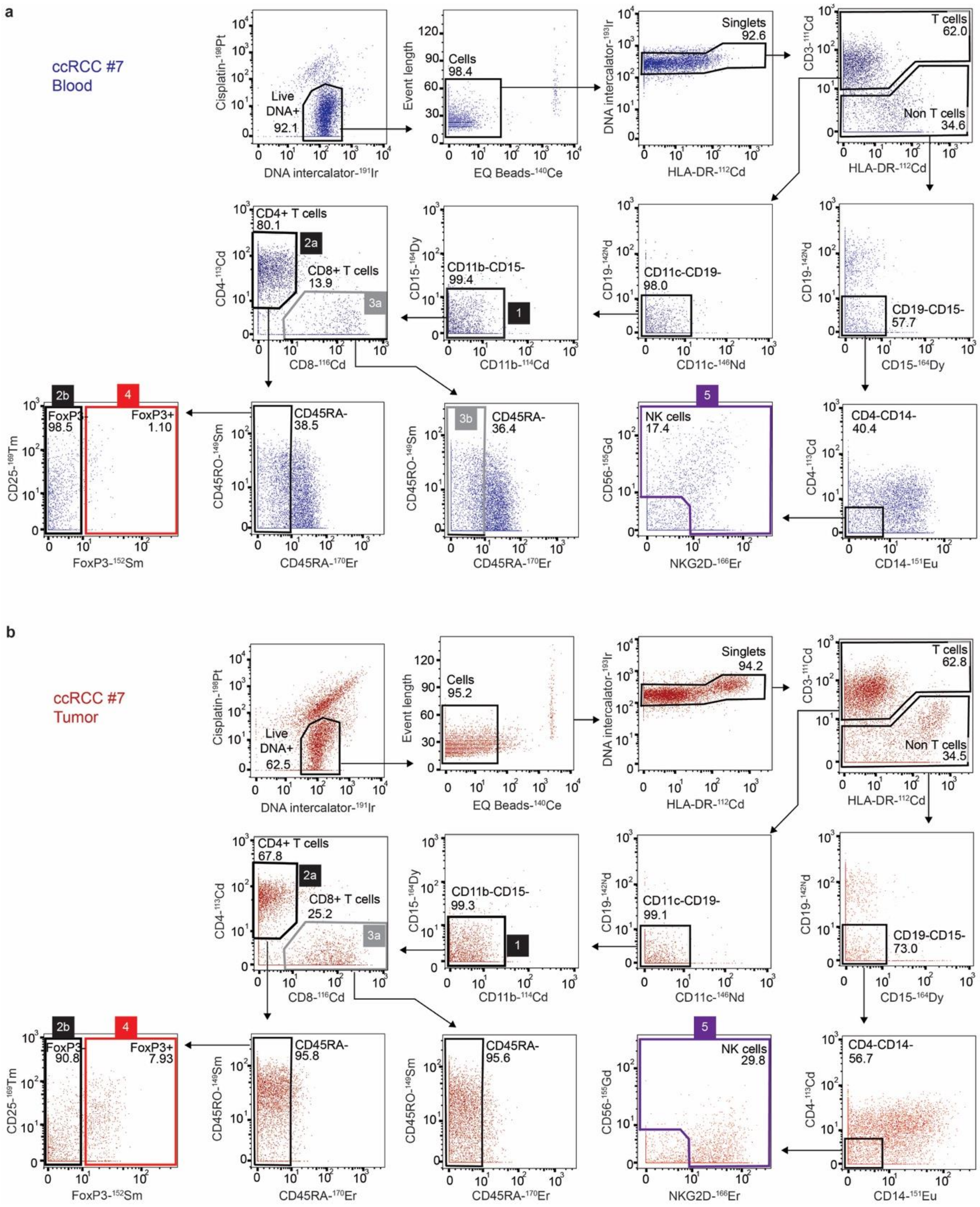

Supplementary Figure 1 – Biaxial gating strategy to identify T cells and NK cells. Prior to biaxial gating displayed here, all files were manually debarcoded based on CD45-89Y, CD45-106Cd, and CD45-110Cd tags and any portion of data where signals were variable across time were removed manually. Representative gating strategy for NK and T cell subsets are shown here from a matched blood (a) and tumour (b) ccRCC sample. First, live single cells were identified based on exclusion of beads, exclusion of cisplatin, incorporation of DNA intercalator, and

event length. Unbiased high-dimensional analysis was used to determine the phenotype of T cells and NK cells in our sample set (data not shown), which then guided biaxial gating strategies to identify CD4+ Tregs, CD4+ T cells (non-Tregs), CD8+ T cells, and NK cells. Based on the data, two gating modifications were made to accommodate cells present in the tumour samples that expressed very high levels of HLA-DR in tumour cells (3rd plot shows HLA-DR^{high} cells incorporate slightly more DNA intercalator; 4th plot shows modest spillover from HLA-DR-^{112}Cd into CD3-^{111}Cd when HLA-DR levels were greater than an MMI of 100). T cells were identified as CD3+CD19-CD11c-CD15-CD11b- and further divided into CD4+ and CD8+ subsets; for analyses, CD45RA+ cells were excluded, and CD4+ T cells were broken down into FoxP3- non-Tregs and FoxP3+ Tregs. Cells from Gate #1 were the input events for T cell analyses (Figure 1a-g, Supplementary Figure 2). Cells from Gate #2a and Gate #3a were the starting populations for TIGIT, CD96, and PD-1 biaxial gating on tumour samples for correlative analysis (Fig 1h). As <5% of tumour T cells were CD45RA+ and >70% of blood T cells were CD45RA+, CD45RA+ T cells were excluded to make comparisons between blood and tumour samples with respect to single and dual expression of TIGIT, CD96, and CD226; thus, events from Gates #2b, #3b, and #4 were used. Based on UMAP analysis of all live cells (data not shown), NK cells were biaxially identified as CD3-CD19-CD15-CD14-CD4- and CD56+ and/or NKG2D+; events from Gate #5 were used for all NK analysis.

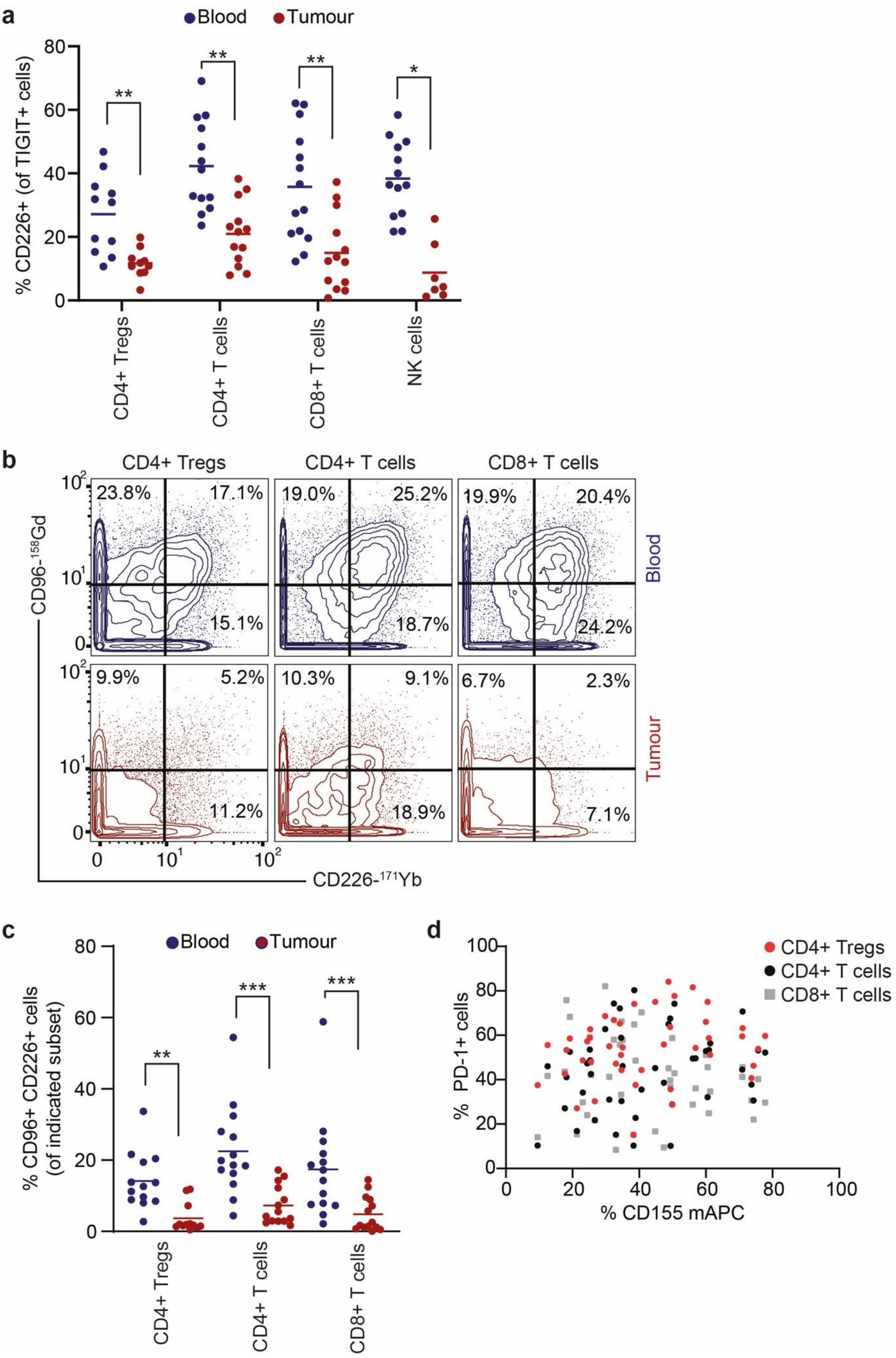
a
Blood
Tumour
% CD226+ (of TIGIT+ cells)
80
60
40
20
0
**
**
**
*
CD4+ Tregs
CD4+ T cells
CD8+ T cells
NK cells
b
CD4+ Tregs
CD4+ T cells
CD8+ T cells
CD96-158Gd
CD226-171Yb
23.8%
17.1%
15.1%
19.0%
25.2%
18.7%
19.9%
20.4%
24.2%
Blood
9.9%
5.2%
11.2%
10.3%
9.1%
18.9%
6.7%
2.3%
7.1%
Tumour
c
Blood
Tumour
% CD96+ CD226+ cells (of indicated subset)
**
***
***
CD4+ Tregs
CD4+ T cells
CD8+ T cells
d
CD4+ Tregs
CD4+ T cells
CD8+ T cells
% PD-1+ cells
% CD155 mAPC
0
20
40
60
80
100

Supplementary Figure 2 – Additional T cell CyTOF data. (a) Mean frequency of CD226 expression within TIGIT+ CD4+ Tregs, CD4+ T cells, CD8+ T cells, or NK cells. *n*=14 matched samples (data excluded where <50 events in parent populations). (b) Co-expression of CD96 and CD226 was investigated as in Figure 1b. (c) Mean frequency of cells co-expressing CD96 and CD226, analysed as in Figure 1c. *n*=14 matched samples (data excluded where <50 events in parent populations). Non-parametric two-tailed matched-pairs Wilcoxon tests were used to determine differences between PBMCs and DTCs (* = ≤0.05, ** = ≤0.01, *** = ≤0.001). (d) Scatter plot displaying the correlation between the frequency of CD155 expression on myeloid antigen-presenting cells (mAPC) with the frequency of PD-1+ CD4+ Tregs (red circles), CD4+ T cells (black circles), or CD8+ T cells (gray squares) in the tumour microenvironment (*n*=44). Source data are provided as a Source Data File.

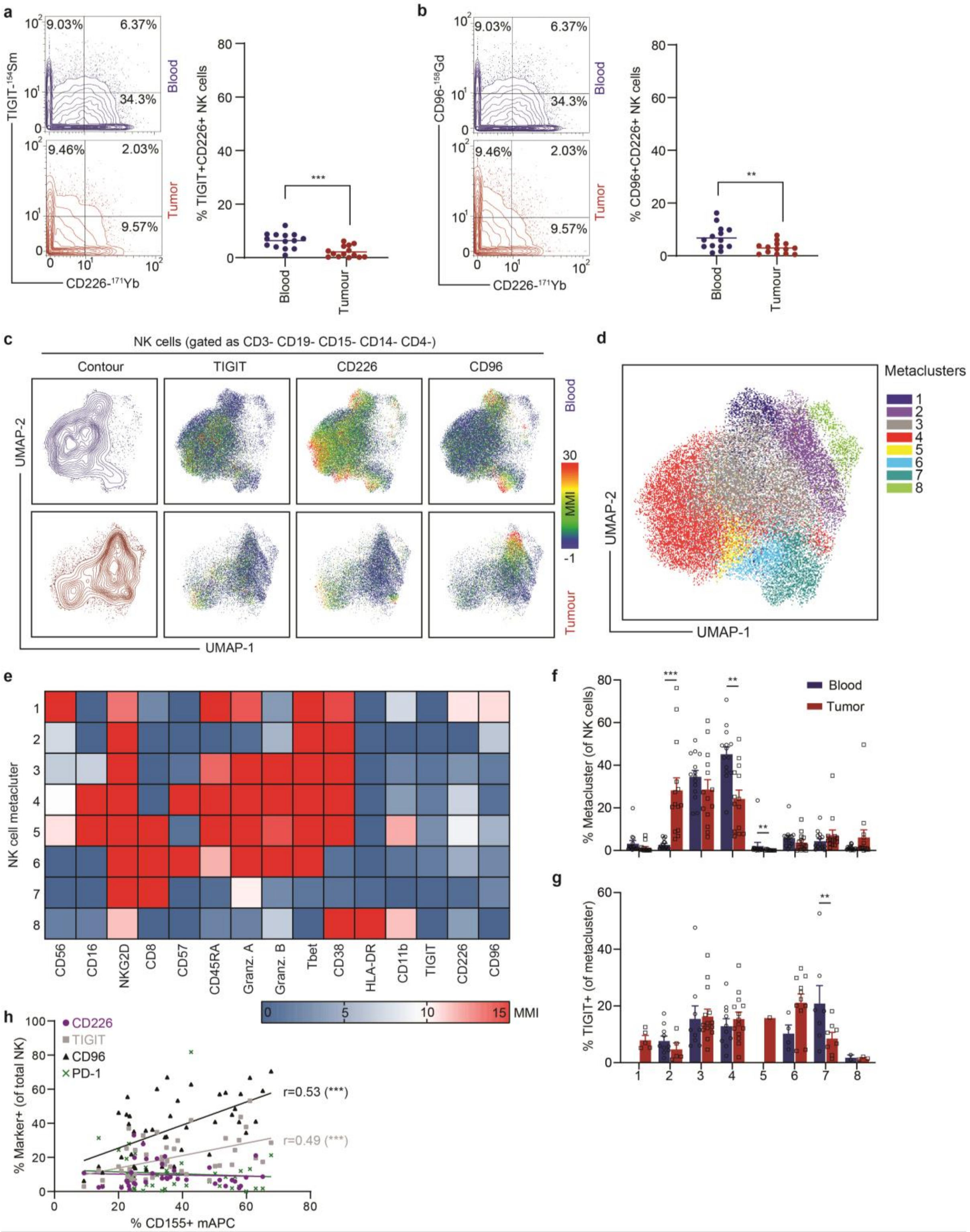

Supplementary Figure 3 – CyTOF analysis of TIGIT and CD226/CD96 expression on NK cells in matched peripheral blood and dissociated tumour cell samples from renal and lung cancer patients. (a) Co-expression of TIGIT and CD226 was investigated on NK cells from the blood (blue) and tumour (red). Plots on the left display and show the frequency of single or co-expression on concatenated files of the matched blood or tumour samples. Graph on the right shows the mean frequency of co-expressing NK cells from each donor. *n*=14 matched samples. (b)

Frequency of NK cells co-expressing CD96 and CD226, analysed the same as in a. *n*=14 matched samples. (c) UMAP analysis performed on NK cells from matched blood and tumour samples (*n*=14). Samples with greater than 3,000 NK cell events (*n*=12) were downsampled to 3,000 events prior to concatenation. UMAP projections show concatenated NK cells from the blood (top; *n*=35,156 events) or tumour (bottom; *n*=15,669 events), highlighting contour (left) or median metal intensity (MMI) of TIGIT, CD226, and CD96 (right). (d) FlowSOM metaclusters were created on NK cells concatenated from all matched samples and projected onto the same UMAP as in c. (e) Expression intensity heatmap of the indicated markers for each of the 8 FlowSOM metaclusters in d. Color scale indicates MMI for each marker. (f) Mean frequency (± SEM) of NK cells in each metacluster within blood and tumour for each individual matched sample. *n*=14 matched samples. (g) Mean frequency (± SEM) of TIGIT+ events (per biaxial gating) within each metacluster. *n*=14 matched samples (data excluded where <50 events in parent populations). Non-parametric two-tailed matched-pairs Wilcoxon tests were used to determine differences between PBMC and DTC (In a, b, f & g; * = ≤0.05, ** = ≤0.01, *** = ≤0.001). (h) Non-parametric Spearman's correlation of the frequency of CD155 expression on myeloid antigen-presenting cells (mAPC) with the frequency of CD226+ (purple circles), CD96+ (black triangles), TIGIT+ (gray squares) and PD-1+ (green crosses) NK cells in the tumour microenvironment (*n*=44, no PBMC data included in analysis). Linear regression line and Spearman rho (r_s) are shown for correlations that were significant (two-tailed; p<0.05, indicated by asterisks after listed rho; *** = ≤0.001). Source data are provided as a Source Data File.

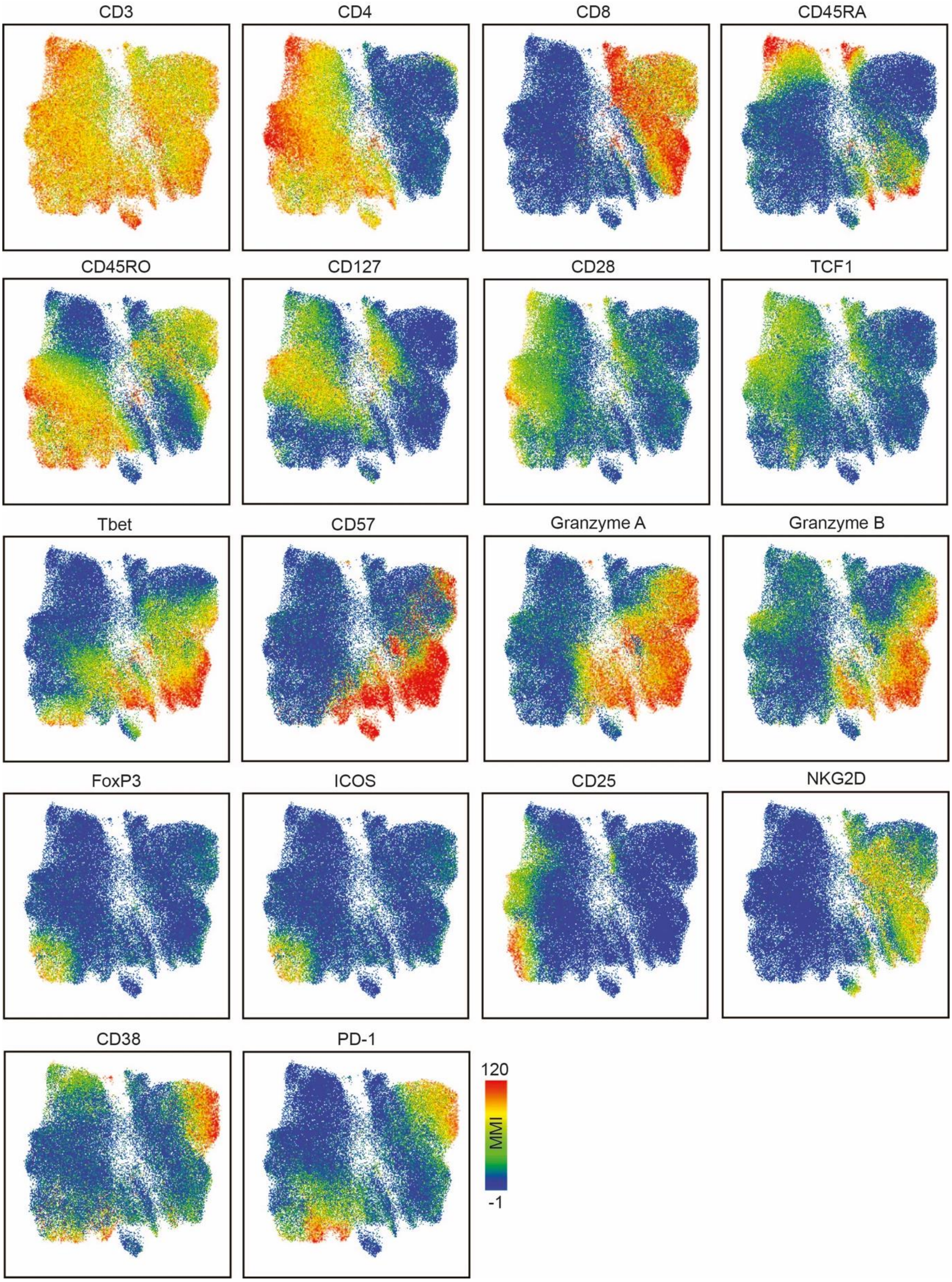

Supplementary Figure 4 – Protein expression patterns on T cell UMAP.

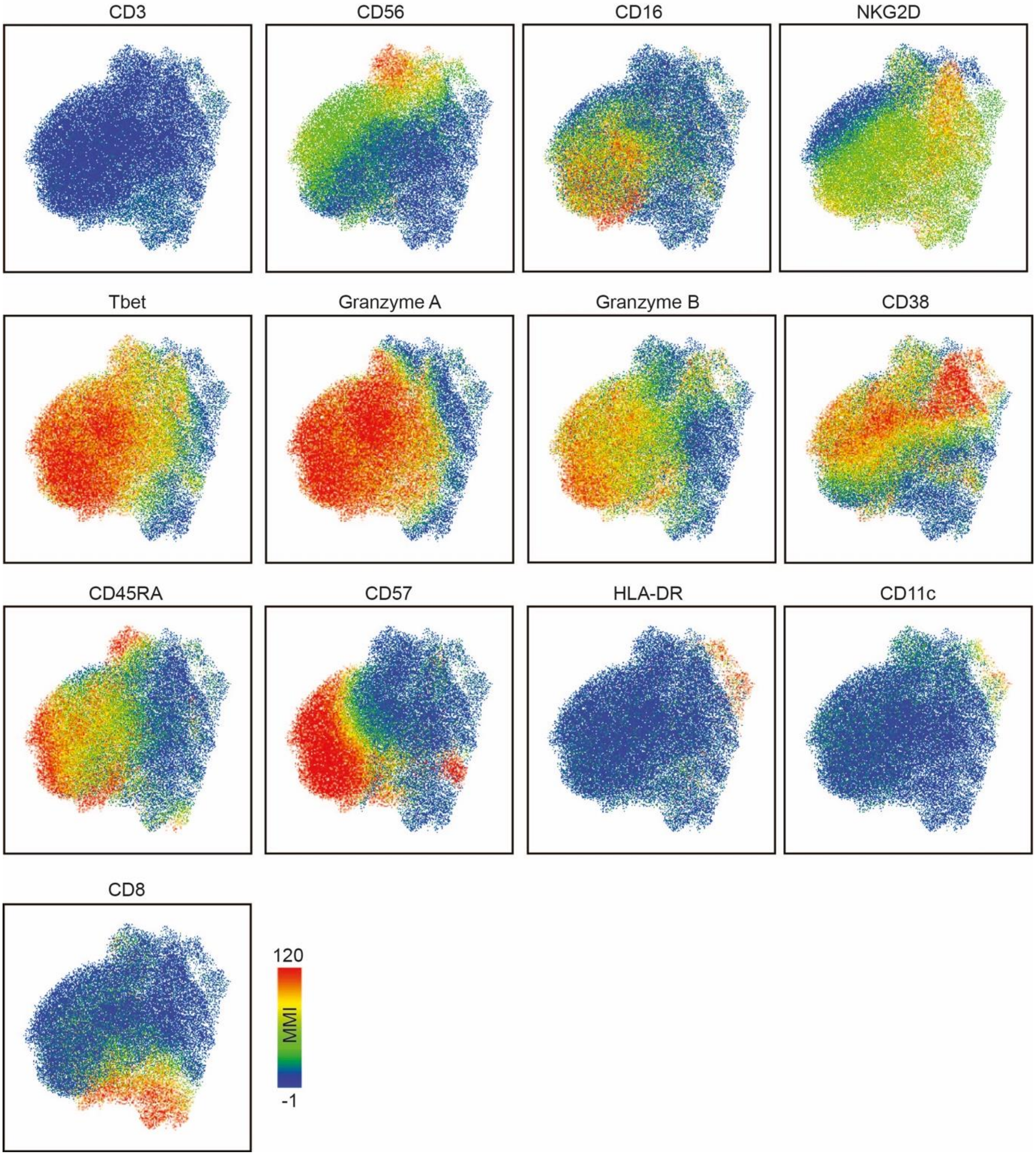

Supplementary Figure 5 – Protein expression patterns on NK cell UMAP.

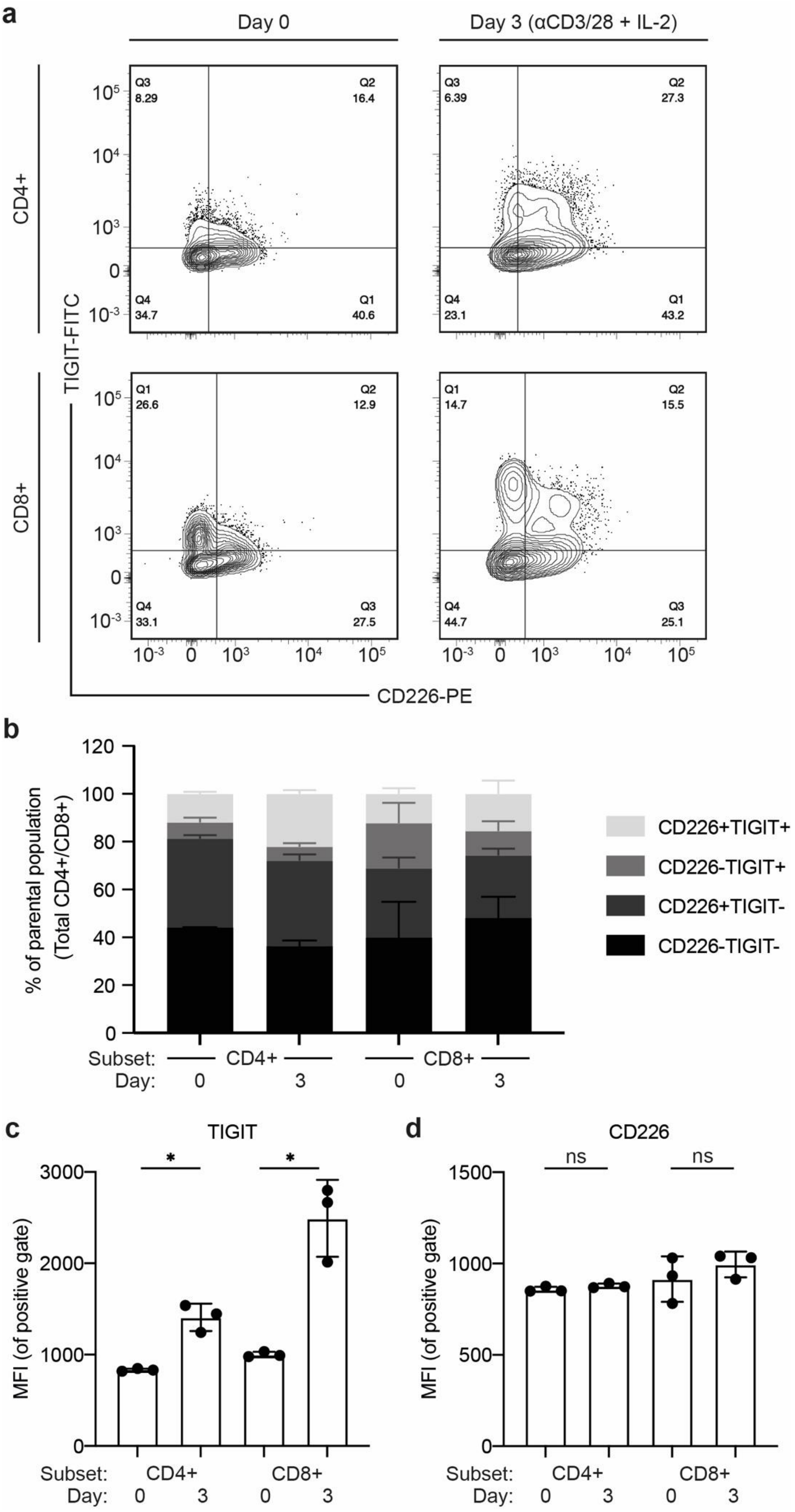
a
Day 0
Day 3 (αCD3/28 + IL-2)
CD4+
CD8+
TIGIT-FITC
CD226-PE
Q3 8.29
Q2 16.4
Q4 34.7
Q1 40.6
Q3 6.39
Q2 27.3
Q4 23.1
Q1 43.2
Q1 26.6
Q2 12.9
Q4 33.1
Q3 27.5
Q1 14.7
Q2 15.5
Q4 44.7
Q3 25.1
b
% of parental population (Total CD4+/CD8+)
CD226+TIGIT+
CD226-TIGIT+
CD226+TIGIT-
CD226-TIGIT-
Subset: CD4+ CD8+
Day: 0 3 0 3
c
TIGIT
MFI (of positive gate)
*
*
d
CD226
ns
ns

Supplementary Figure 6 – Characterisation of primary T cells used in the manuscript. (a) Biaxial plots showing TIGIT and CD226 expression on primary CD4+ or CD8+ T cells on the day of isolation or following 3 days of stimulation on plates coated with anti-CD3 and anti-CD28 mAbs with IL-2, as indicated. (b) Stacked bar chart showing the proportion of cells in the indicated populations. Bars represent the mean ±S.D**.** (c,d) Median fluorescent intensity (MFI; ±S.D) of TIGIT (c) and CD226 (d) in their respective positive gates (as set by isotype controls). Statistical comparisons are made with two-tailed paired T tests within each subset (ns = not significant, * ≦ 0.05; *n* = 3 individual blood donors). Source data are provided as a Source Data File.

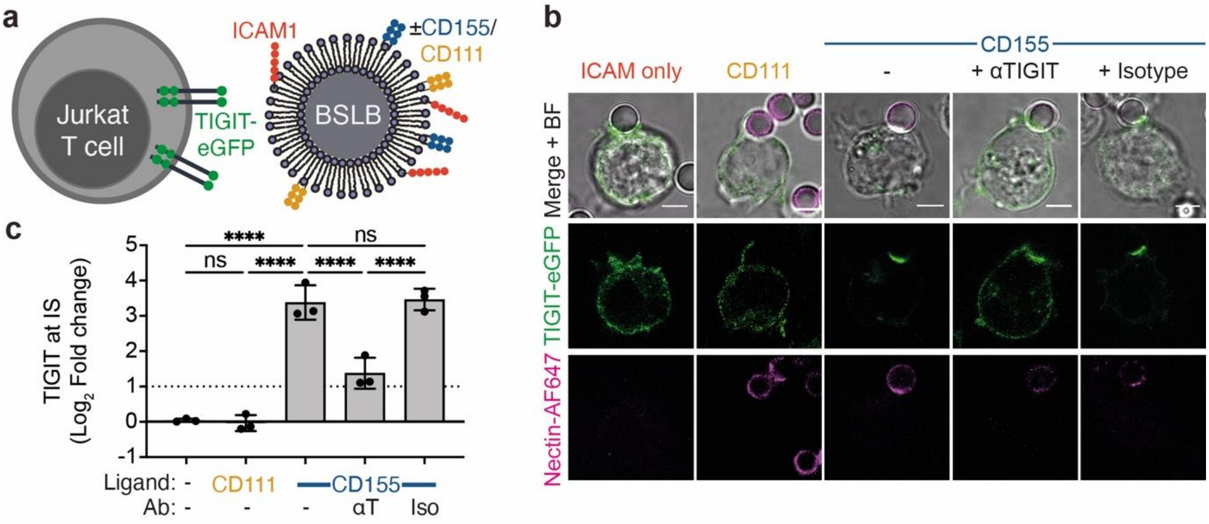

Supplementary Figure 7 – Bead-supported lipid bilayers (BSLBs) phenocopy observations with cell-cell conjugates. (a) Schematic depicting the BSLBs, used as a surrogate for an Antigen Presenting Cell or a tumour cell, containing mobile nectin ligands (CD111 or CD155), and the adhesion molecule ICAM-1. (b) Confocal microscopy images showing the spatial distribution of TIGIT-GFP (green) on the surface of Jurkat T cells conjugated to BSLBs for 20 mins loaded with Alexa Fluor 647-labelled his-tagged ligands CD111 or CD155 (400 molecules/μm^2; magenta), as indicated above and ICAM-1 (100 molecules/μm^2). Cells pre-treated with an antagonistic TIGIT antibody, or an isotype matched control are shown, as indicated. A merged image with the respective BF image is also provided. (c) Mean $\log_2$ fold change (±S.D., n = 3 independent experiments) increases in synaptic TIGIT in Jurkat cells depicted in b. P values from a one-way ANOVA are displayed (ns = not significant, **** ≦ 0.0001). All scale bars = 5 μm. Source data are provided as a Source Data File.

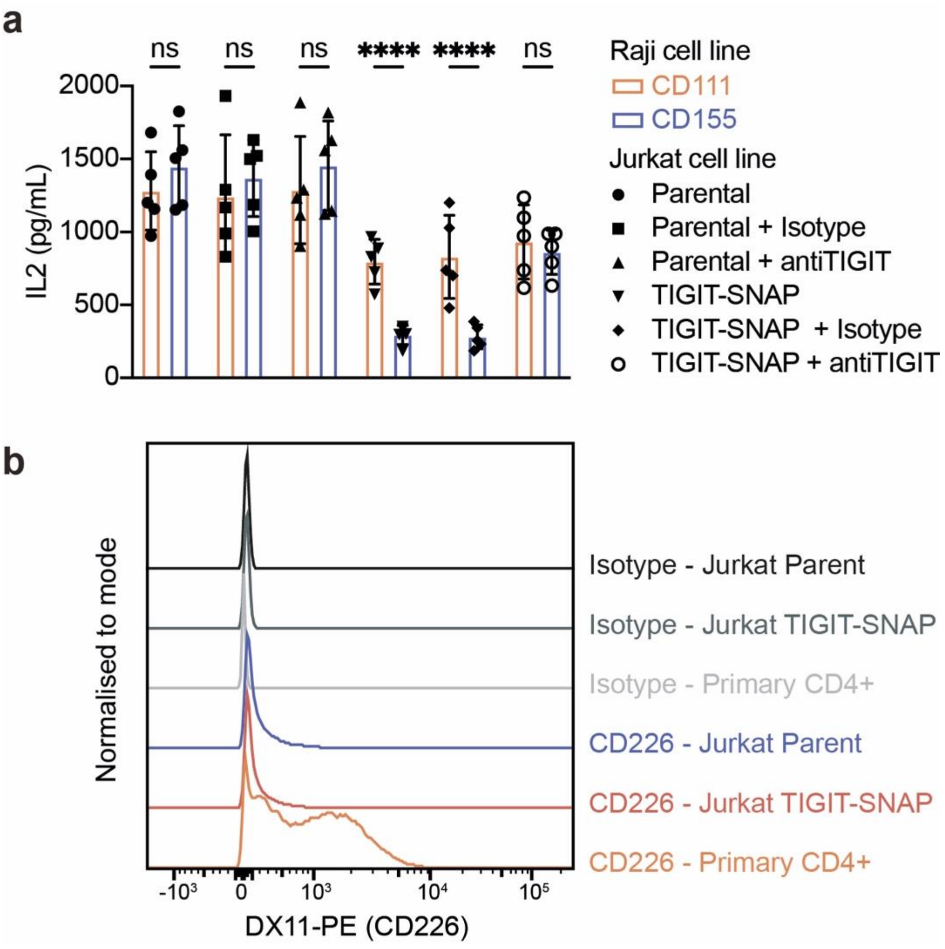

Supplementary Figure 8 – Supplementary ELISA and flow cytometry data to support Jurkat-Raji co-culture models. (a) ELISA data showing the absolute amount of IL-2 released from either parental or TIGIT-SNAP-expressing Jurkat cells after co-incubation with SEE-pulsed Raji cells (coloured for CD111- or CD155-expressing, as indicated) for 6 hours. Bars represent the mean (±S.D.), *n*=5 biological repeats. Cells pre-incubated with an antagonistic TIGIT antibody or an isotype-matched control are shown, as indicated. A 2-way ANOVA, with Šídák's multiple comparisons test, was used for statistical analysis (ns = not significant, **** = P<0.0001). (b) Flow cytometry analysis showing the relative expression of CD226 in different Jurkat cell lines and primary CD4+ T cells (as indicated to the right), through DX11-PE antibody staining and isotype-matched controls. Source data are provided as a Source Data File.

a

b

c

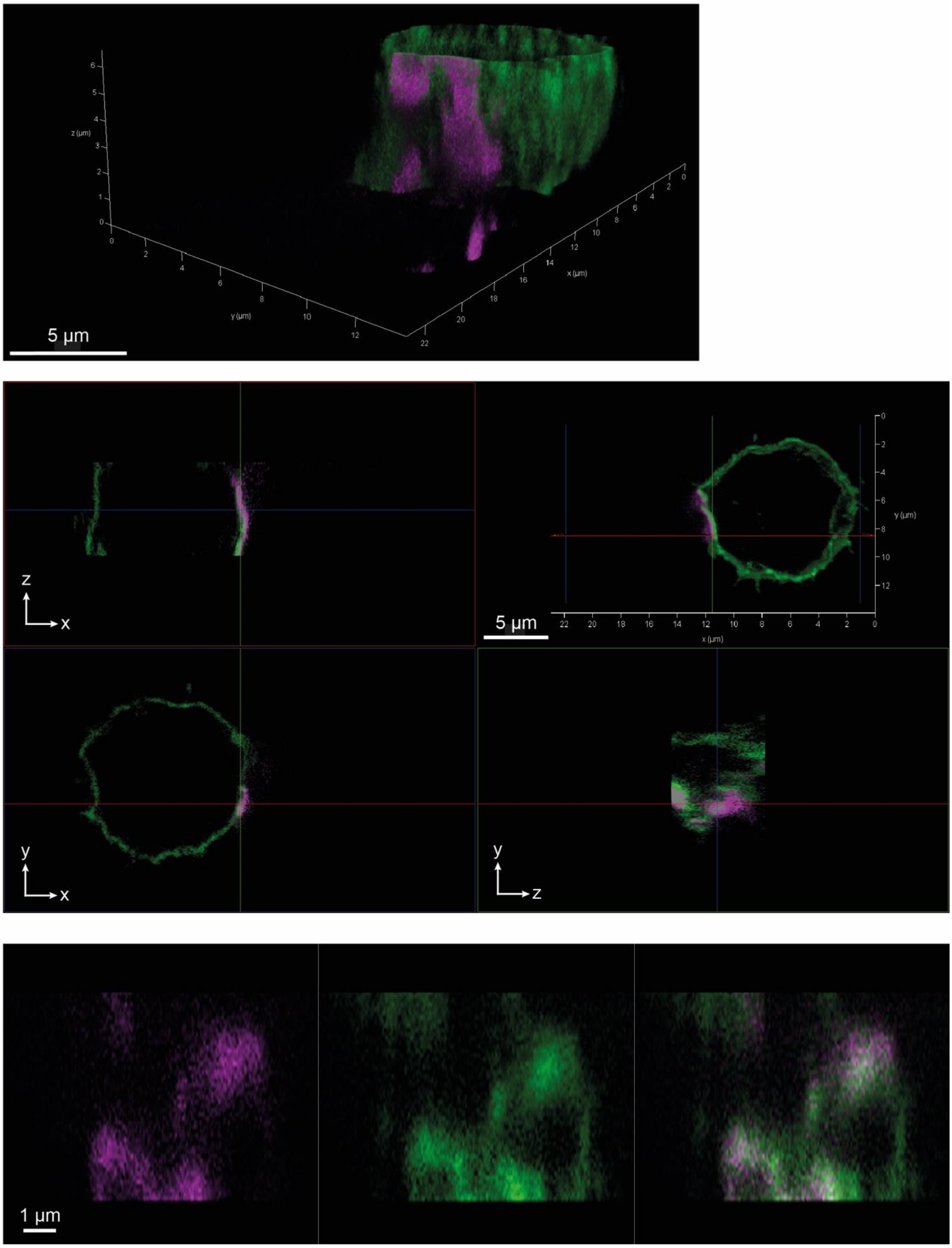

Supplementary Figure 9 – Nanoscale clustering of TIGIT-CD155 as shown by 3D τSTED imaging of a Jurkat-TIGIT-SNAP-Raji-CD155 conjugate. (a) 3D volume of a Jurkat TIGIT-SNAP cell (labelled with the SNAP label TMR-STAR; magenta) conjugated to a Raji-CD155 (labelled by a V5/Secondary-AF514 immunostain; green), showing the 3-dimensional organisation of the two molecules on the cells after conjugation for 10 mins. (b) Orthogonal slice views of the conjugate with the volume shown in the top right, and xz, xy and zy images of slices along the red (x),

green (y) and blue (z) planes shown in the images. (c) A section clipped from the volume to show the *en face* view of the IS, with TIGIT (magenta), CD155 (green) and a merged view (right) shown.

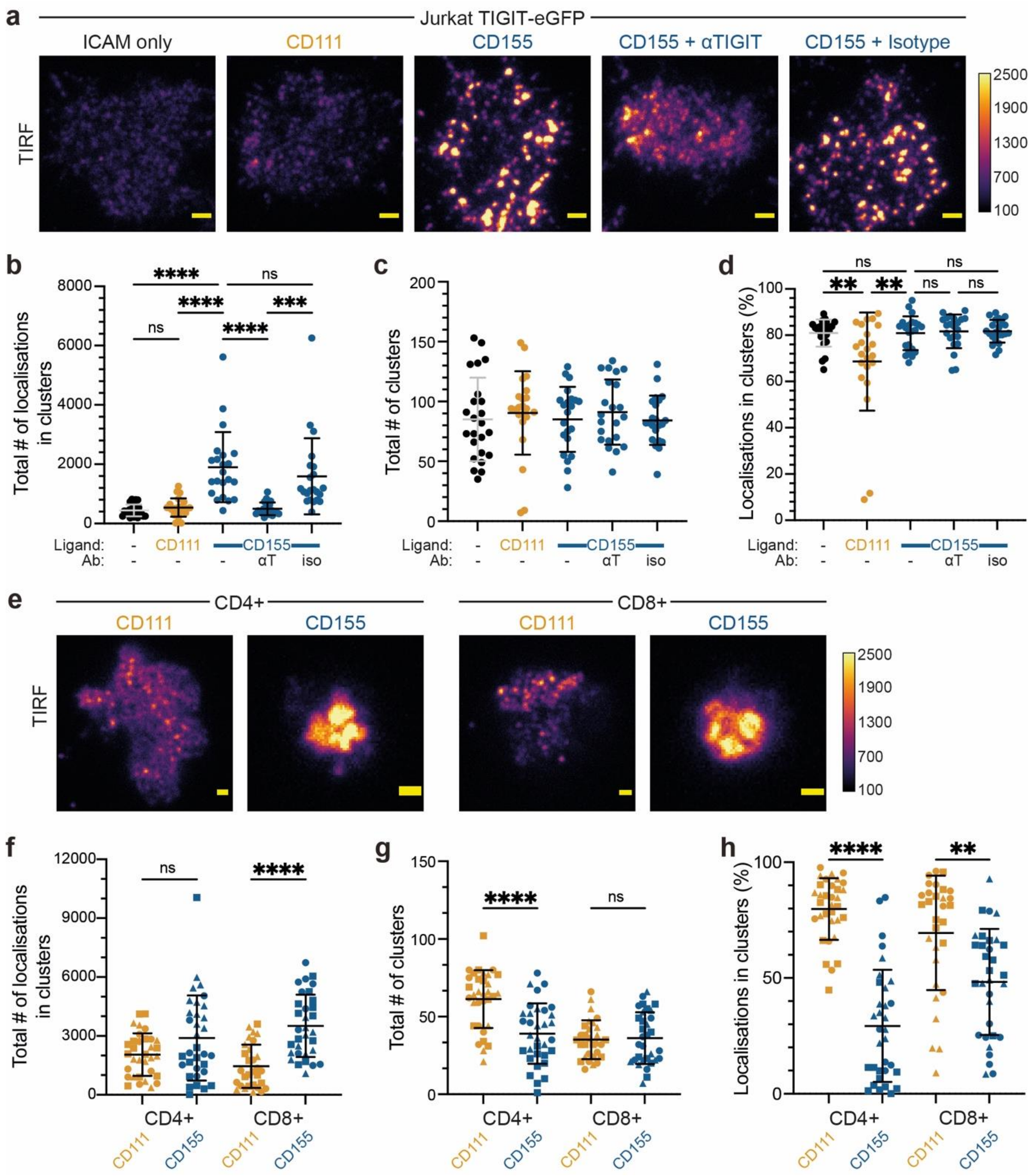

Supplementary Figure 10 – Supplementary dSTORM data showing ligation-induced TIGIT clustering. (a) Equivalent TIRF images for the dSTORM images shown in Figure 3a. Images are acquired with equal settings, and brightness is scaled to allow a fair comparison, with the colour bar displaying arbitrary intensity values provided on the right. Scale bar = 2 μm. (b-d) Additional quantification from the clustering analysis of dSTORM imaging in Jurkat cells, *n* = ≥22 cells per condition, representative of 3 independent experiments. Graphs show the mean (±S.D.) total number of events deemed to be within clusters (b), the total number of clusters (c) and the percentage of all events to be deemed within clusters (d), per region of a cell analysed. (e) Equivalent TIRF images for the dSTORM images shown in Figure 3f-g and coloured as in a. Scale bar = 1 μm. (f-h) Additional quantification from the clustering analysis of dSTORM imaging in primary peripheral

blood isolated T cells, n = ≥30 cells per condition, representative of 3 donors (labelled by symbol shape). Graphs show the mean (±S.D.) total number of events deemed to be within clusters (f), the total number of clusters (g) and the percentage of all events to be deemed within clusters (h), per region of a cell analysed. Throughout, P values from a one-way ANOVA are displayed (ns = not significant, ** = ≦0.01, *** = ≦0.001 & **** = ≦0.0001). Source data are provided as a Source Data File.

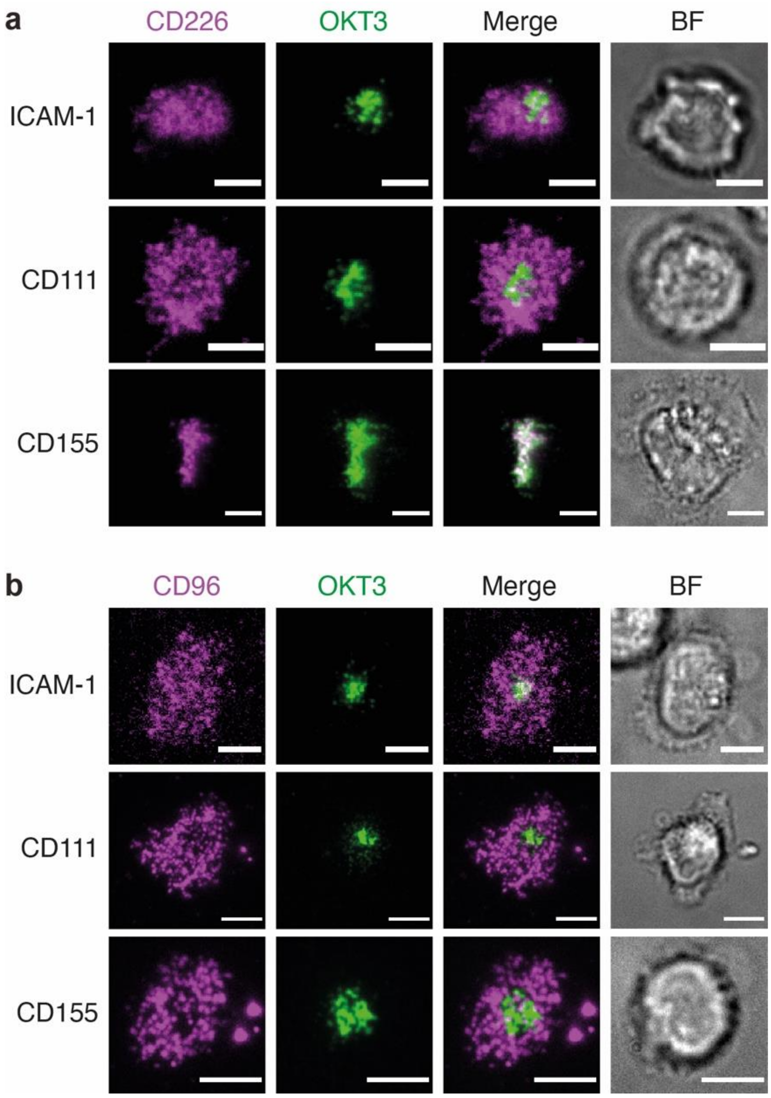

Supplementary Figure 11 – CD226 but not CD96 shows similar co-clustering with TCR upon ligation. (a, b) Representative TIRF microscopy images showing the relative spatial distribution of CD226 (a) or CD96 (b) and the TCR at the IS of primary CD4+ T cells that have interacted with PLBs loaded with ICAM-1 (100 molecules/μm^2), and either CD111 or CD155 (400 molecules/μm^2) for 10 mins. Images are representative of at least 3 independent donors. Respective brightfield images are also shown. Scale bars = 5 μm.

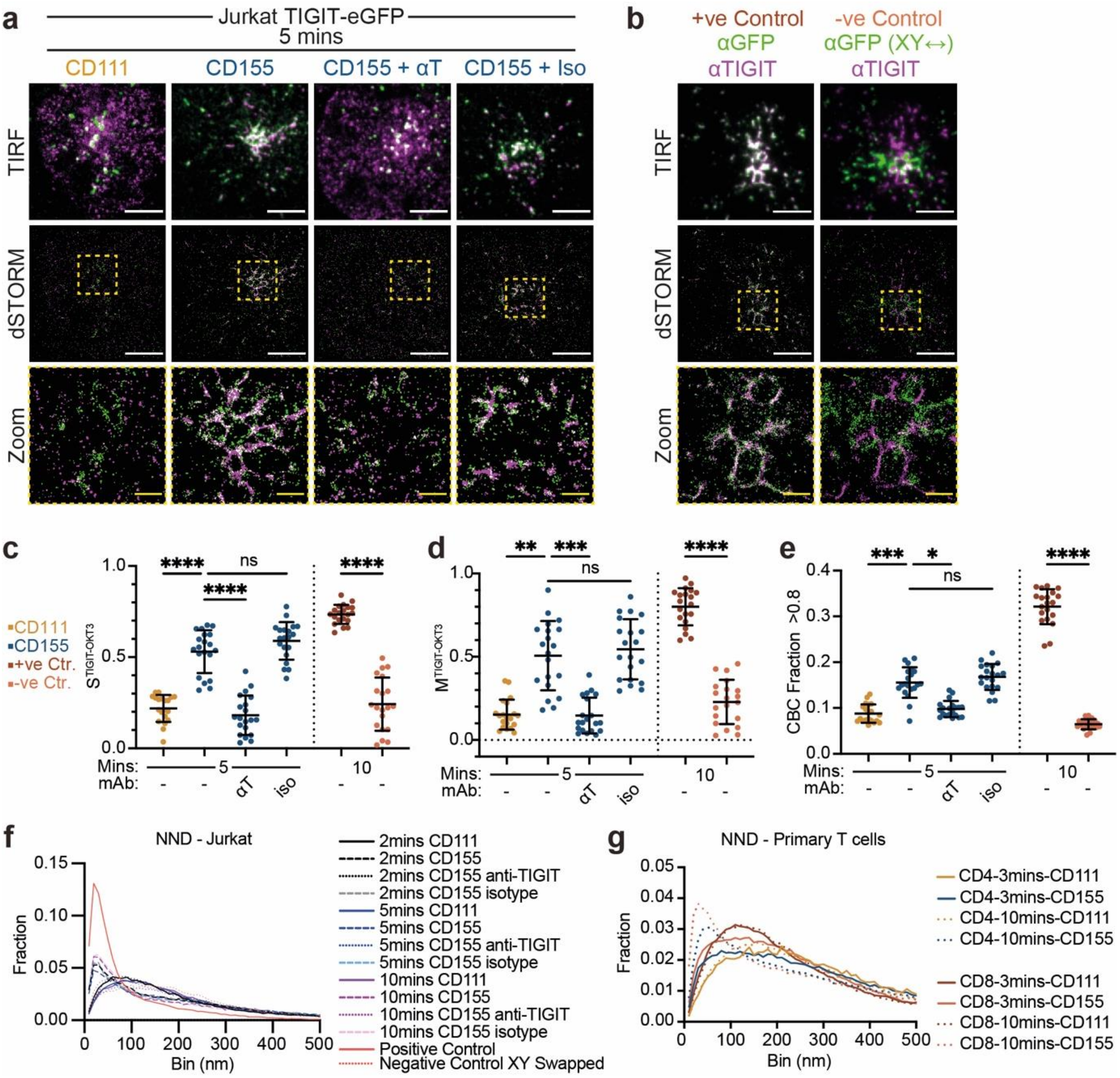

Supplementary Figure 12 - Supplementary two-colour dSTORM data showing co-clustering of TIGIT and TCR clusters. (a) Two-colour dSTORM imaging of TIGIT-GFP (magenta) in Jurkat cells that have interacted with PLBs loaded with ICAM-1 (100 molecules/μm^2), the indicated nectin ligands (400 molecules/μm^2) and directly labelled, mono-biotinylated stimulatory TCR antibody OKT3 (green) for 5 mins. Cells pre-incubated with an antagonistic TIGIT antibody (αT) or an isotype-matched control (iso) are shown, as indicated. Representative TIRF and dSTORM images are shown in the top and middle rows, respectively. Scale bar = 5 μm. Zoomed regions (5 μm x 5 μm; dashed yellow boxes) are displayed below with the scale bar = 1 μm. (b) Two-colour dSTORM imaging of TIGIT-GFP in Jurkat cells labelled with both an anti-GFP nanobody (green) and a TIGIT-directed mAb (magenta), with the negative control representing the same data but with the XY coordinates of one channel being swapped. Cells interacted with PLBs, as in a, for 10 mins. Scale bar = 5 μm. Zoomed regions (5 μm x 5 μm; dashed yellow boxes) are displayed below with the scale bar = 1 μm. (c-e) Quantitative analysis of the colocalisation between TIGIT and TCR in Jurkat cells as shown in a; *n* = ≥20 cells per condition, representative of 3 independent experiments, with ANOVA being used for statistical analysis. Positive and negative controls depicted in b are also shown.

Mean (±S.D.) Spearman rank correlations (c) and Mander's coefficients (d) for TIGIT-OKT3 localisations across each cell. (e) Mean (±S.D.) fraction of localisations that have a score of >0.8 from a coordinate-based colocalisation analysis across single cells. (f-g) Histograms depicting the distribution of nearest neighbour distances (NND) measured between localisations of two colours, in the indicated conditions, for both Jurkat (f) and primary T cells (g). Ordinary one-way ANOVA was used for statistical analysis of Spearman's correlations and Kruskal-Wallis tests with Dunn's multiple comparisons for Mander's correlations and CBC analysis (ns = not significant, * = ≤0.05, ** = ≤0.01, *** = ≤0.001, **** = ≤0.0001). Source data are provided as a Source Data File.

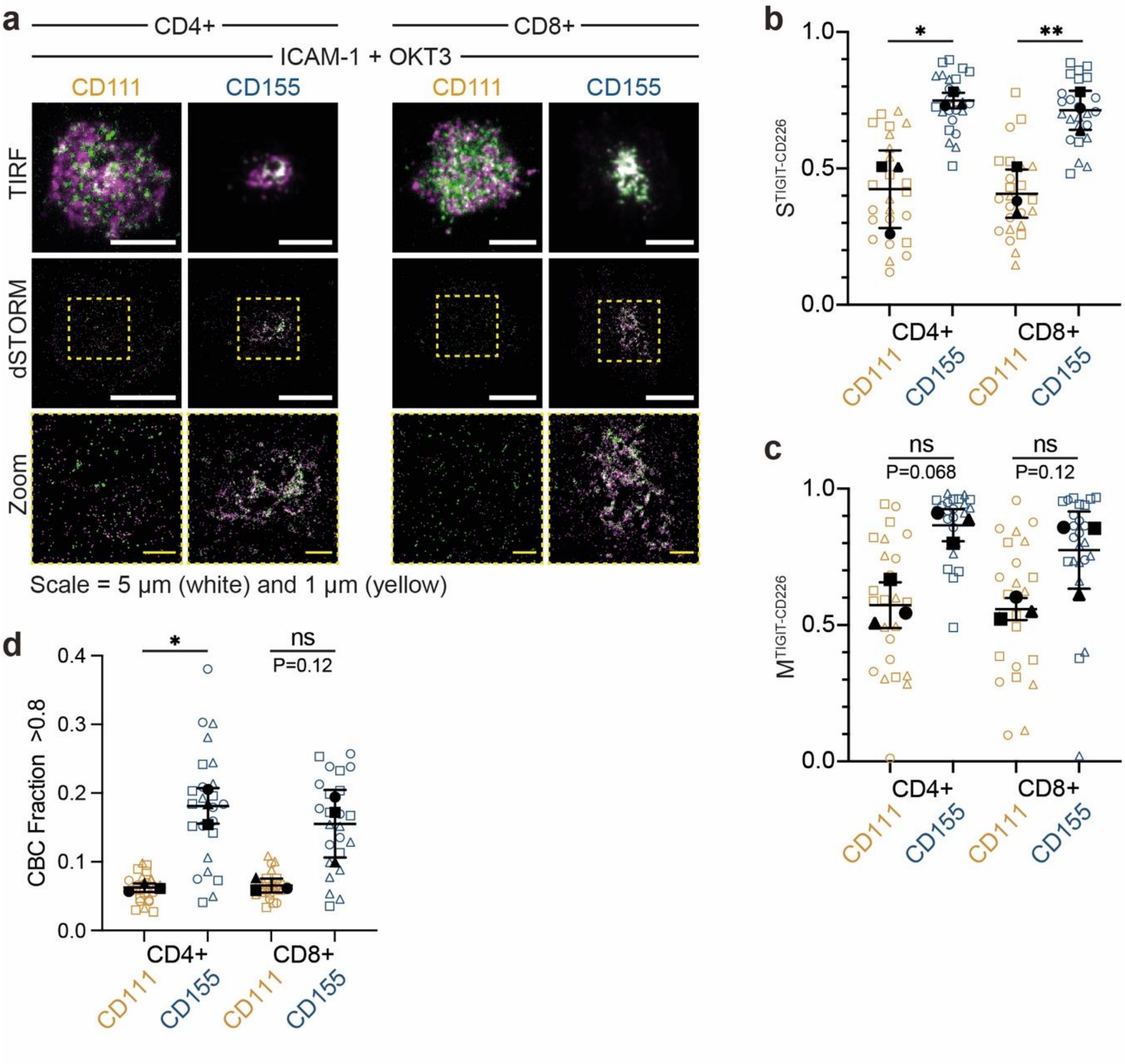

Supplementary Figure 13 - Super resolution microscopy reveals the nanoscale proximity of TIGIT and CD226 clusters. (a) Two-colour TIRF and dSTORM imaging of TIGIT (magenta) and CD226 (green) in primary CD4+ or CD8+ T cells that have interacted with PLBs loaded with ICAM-1 (100 molecules/μm^2), the indicated nectin ligands (400 molecules/μm^2) and mono-biotinylated stimulatory TCR antibody OKT3 for 10 minutes. Representative TIRF and dSTORM images are shown in the top and middle rows, respectively. Zoomed regions (dashed yellow boxes) are displayed below. (b-d) Quantitative analysis of the colocalisation between TIGIT and CD226 in cells as shown in a; *n* = 24 cells per condition (8 each from 3 individual blood donors, represented by a different shape), with two-tailed paired T tests being used for statistical analysis on the biological means within each subset (filled shapes; ns = not significant, * = ≤0.05, ** = ≤0.01). Lines represent the mean (±S.D. of biological replicates) Spearman rank correlations (b) and Mander's coefficients (c) for TIGIT-CD226 localisations across each cell. (d) Mean (±S.D. of biological replicates) fraction of localisations that have a score of >0.8 from a coordinate-based colocalisation analysis across single cells. Source data are provided as a Source Data File.

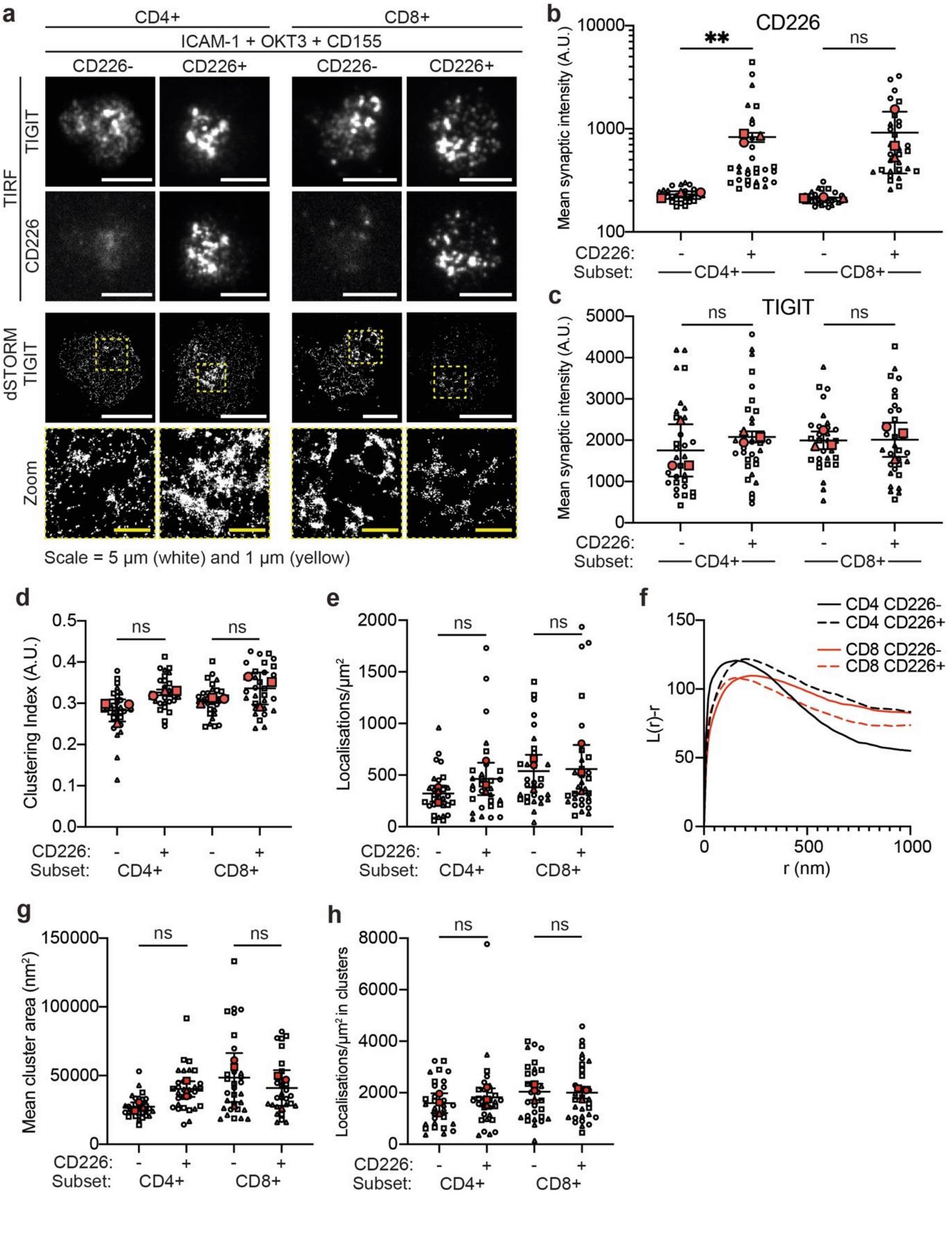

Supplementary Figure 14 – CD226 does not significantly affect TIGIT clustering. (a) TIRF imaging of TIGIT and CD226 (top two rows) in primary CD4+ or CD8+ T cells that have interacted with PLBs loaded with ICAM-1 (100 molecules/µm^2), CD155 (400 molecules/µm^2) and mono-biotinylated stimulatory TCR antibody OKT3 for 10 minutes. TIGIT$^+$CD226$^-$ and TIGIT$^+$CD226$^+$ subsets were delineated by their staining intensities and displayed in the indicated columns. Single

colour dSTORM was performed on the corresponding TIGIT channel (third row) with zoomed regions (dashed yellow boxes) displayed below. Quantification of synaptic CD226 (b) and TIGIT (c), and TIGIT clustering index (d) measured from TIRF images as shown in a. (e-h) Quantitative analysis of the single molecule localisation images shown in a. (e) Density of TIGIT localisations within analysed regions. Each datapoint represents a 9 μm^2 region from a single cell. (f) Mean Ripley's H function, at different clustering radii, for all cells analysed within each condition. (g) Cluster area with each datapoint (hollow shapes) representing the mean cluster size per region of a single cell (±S.D. of biological replicates, red shapes). (h) Density of events within clusters for each condition (±S.D.). In all panels, lines depict the mean (±S.D.) of biological replicates and paired two-tailed T tests were used for statistical comparisons within each T cell subset (*n* = 30 cells per condition, 10 each from 3 individual blood donors represented by different shapes; ns = not significant, ** = ≤0.01). Source data are provided as a Source Data File.

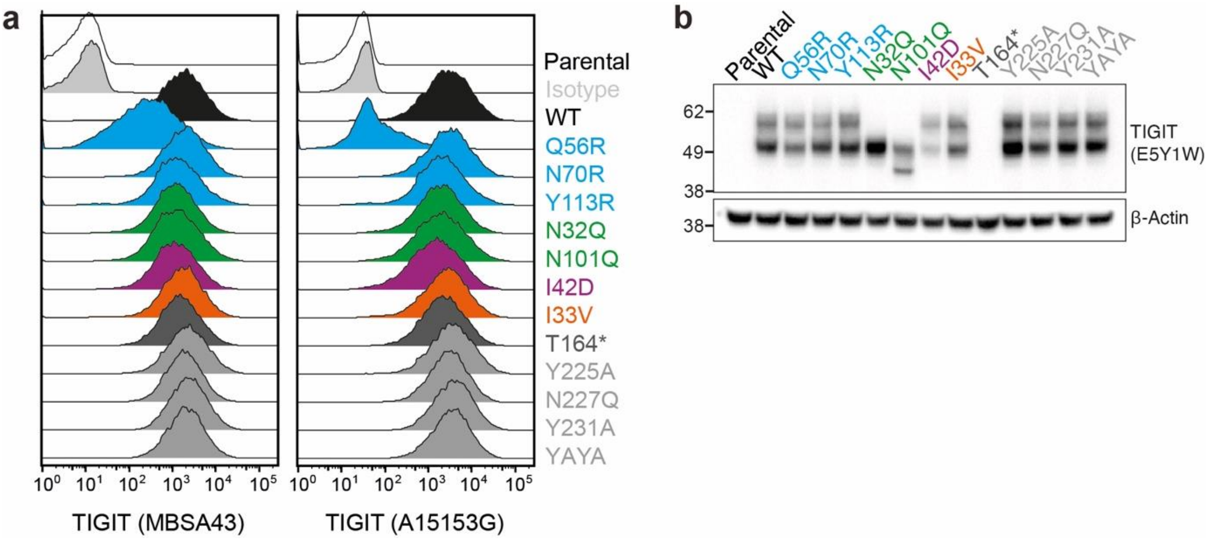

Supplementary Figure 15 – Characterisation of WT and mutant TIGIT-SNAP expression in Jurkat T cell lines. (a) Flow cytometric analysis showing the relative expression of TIGIT-SNAP constructs transduced into Jurkat T cells, through staining with two different TIGIT-directed mAbs (clones MBSA43 and A15153G). (b) Western blot showing the TIGIT-SNAP expression in Jurkat T cell lines, stained with a TIGIT mAb (clone E5Y1W). A loading control blot for β-Actin is also shown below from the same lysates. Data are representative of 2 independent experiments.

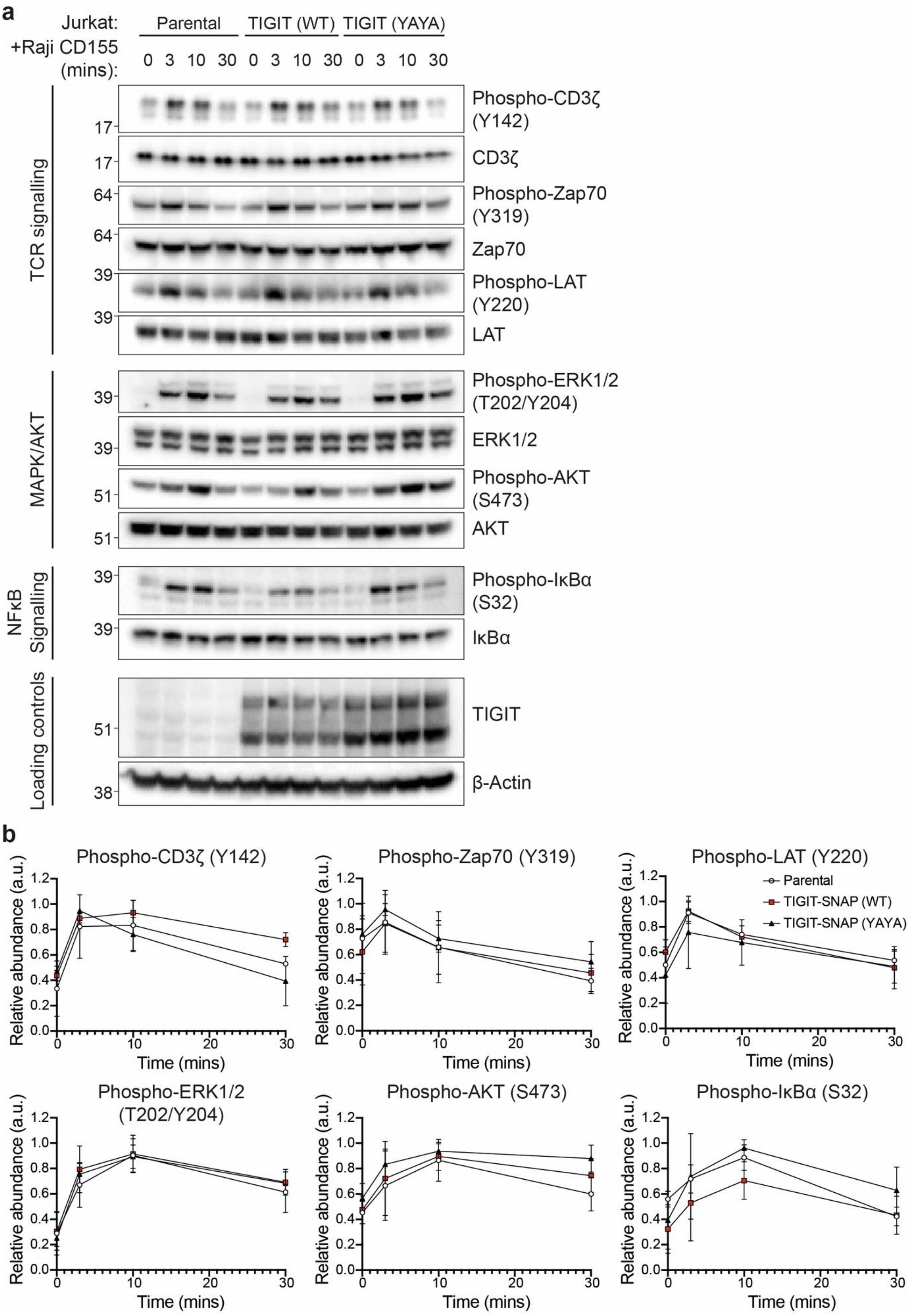

Supplementary Figure 16 – Western blotting reveal TIGIT does not affect TCR, MAPK, AKT and NFκB signalling in Jurkat cells. (a) Images from Western blots of

the indicated proteins and phosphoproteins in Jurkat-Raji conjugates (E:T ratio 3:1). Parental, TIGIT-SNAP (WT) and TIGIT-SNAP (Y225A/Y231A; YAYA) expressing Jurkat cells were conjugated to SEE-pulsed Raji CD155 cells for the indicated times. MW markers from a control protein ladder are shown on the left for reference and represent kDa. (b) Normalised quantification of each phosphoprotein at the indicated timepoints for each Jurkat cell line (depicted with open circles for parental, red squares for WT and black triangles for YAYA). Relative intensities (±S.D.) were normalised to β-Actin on the same membrane. *n*=2 for phospho-CD3ζ and *n*=3 biological repeats for all others. Source data are provided as a Source Data File.

Supplementary Tables

Supplementary Table 1 – CyTOF study sample information.

Primary indication	Age (Average)	Gender	Race	Treatment Status	Clinical Stage	Smoking history	Alcohol History	Matched sample
ccRCC (n=13)	67	M = 7	Caucasian = 7	Naive = 13	I = 5	Never used = 4	Never used = 2	Matched = 8
					II = 3	Previous use = 3	Past use = 0	Not matched = 5
					III = 2	Current use = 3	Current use = 3	
		F = 6	Unknown = 7		IV = 1	Unknown = 3	Unknown = 8	
					Unknown = 2			
Lung SCC (n=7)	55	M = 4	Caucasian = 7	Naïve = 7	I = 2	Never used = 1	Never used = 1	Matched = 2
					II = 4	Previous use = 3	Past use = 0	Not matched = 5
		F = 3			III = 1	Current use = 1	Current use = 2	
						Unknown = 2	Unknown = 4	
Lung NSCLC (n=23)	64	M = 12	Caucasian = 20	Naïve = 23	I = 1	Never used = 8	Never used = 0	Matched = 4
			Black = 1		II = 10	Previous use = 3	Past use = 0	Not matched = 19
					III = 9	Current use = 6	Current use = 9	
		F = 11	Unknown = 2		IV = 2	Unknown = 6	Unknown = 14	
					Unknown = 1			

Supplementary Table 2 – CyTOF Panel Information.

Metal Tag	Target	Clone	Conjugation (purified mAb vendor)	Dilution*
89Y	CD45	HI30	Fluidigm	1:100
106Cd	CD45	HI30	In house (BioLegend)	1:100
110Cd	CD45	HI30	In house (BioLegend)	1:200
111Cd	CD3	UCHT1	In house (BioLegend)	1:50
112Cd	HLA-DR	L243	In house (BioLegend)	1:200
113Cd	CD4	RPA-T4	In house (BioLegend)	1:100
114Cd	CD11b	ICRF44	In house (BioLegend)	1:100
116Cd	CD8a	RPA-T8	In house (BioLegend)	1:100
141Pr	CD28	CD28.2	In house (BioLegend)	1:50
142Nd	CD19	HIB19	Fluidigm	1:200
143Nd	CD127	A019D5	Fluidigm	1:50
n/a	CX3CR1-FITC	2A9-1	n/a (BioLegend)	1:50
144Nd	FITC	FIT22	Fluidigm	1:50
145Nd	TCF1	7F11A10	In house (BioLegend)	1:100
146Nd	CD11c	3.9	Fluidigm	1:100
147Sm	Granzyme A	CB9	In house (BioLegend)	1:100
148Nd	PD-1	EH12.2H7	In house (BioLegend)	1:100
149Sm	CD45RO	UCHL1	Fluidigm	1:100
151Eu	CD14	M5E2	Fluidigm	1:100
152Sm	FoxP3	PCH101	In house (eBioscience)	1:200
153Eu	CD206	15-2	In house (BioLegend)	1:100
154Sm	TIGIT	MBSA43	Fluidigm	1:50
155Gd	CD56	B159	Fluidigm	1:50
156Gd	IFNg	4S.B4	In house (eBioscience)	1:100
158Gd	CD96	In house	In house	1:50
159Tb	PD-L1	29E.2A3	Fluidigm	1:50
160Gd	Tbet	4B10	Fluidigm	1:200
161Dy	CD107a	H4A3	In house (BioLegend)	1:1600
163Dy	CCR8	L263G8	In house (BioLegend)	1:25
164Dy	CD15	W6D3	Fluidigm	1:100
165Ho	CD101	BB27	Fluidigm	1:100
166Er	NKG2D	ON72	Fluidigm	1:50
167Er	CD112	TX31	In house (BioLegend)	1:100
168Er	Ki-67	B56	Fluidigm	1:1600
169Tm	CD25	2A3	Fluidigm	1:400
170Er	CD45RA	HI100	Fluidigm	1:800
171Yb	CD226	DX11	Fluidigm	1:50
172Yb	CD38	HIT2	Fluidigm	1:100
173Yb	Granzyme B	GB11	Fluidigm	1:800
174Yb	CD57	HNK-1	In house (BioLegend)	1:3200
175Lu	CD155	SKII.4	In house (BioLegend)	1:100
176Yb	ICOS	C398.4A	In house (BioLegend)	1:200
Ir191/193	DNA	n/a	Fluidigm	1:2000
198Pt	Cisplatin	n/a	Fluidigm	1:4000
209Bi	CD16	3G8	Fluidigm	1:50

*Note in house conjugated antibodies are at 0.5 mg/mL prior to labelling.

Supplementary Table 3 – Additional information for T cell FlowSOM metaclusters.

			% TIGIT+ in PBMC vs DTC	% Metaclusters in PBMC vs DTC		
			Wilcoxon t-test	Wilcoxon t-test	Spearman's correlation	
**Metacluster**	**Phenotype**	**Defining Markers**	**p**	**p**	**r**	**p**
1	CD4 Tnaive	CD4+CD45RA+TCF1+	**0.0137**	**0.009**	0.2308	0.4265
2	CD4 Tcm	CD4+CD45RO+TCF1+Tbet-GrzA-	0.2166	0.2412	-0.1209	0.6818
3	CD4 Tem (transitional?)	CD4+CD45RO+TCF1+Tbet+GrzA+	0.5000	0.391	0.05292	0.8568
4	CD4 Tfh-like	CD4+CD45RO+PD-1+ICOS+	**0.0420**	**0.0001**	0.5253	0.0567
5	CD4 Tregs	CD4+FoxP3+CD127-CD25+	**0.0039**	0.058	0.274	0.3404
6	CD4 Terminally differentiated	CD4+Tbet+GrzA/B+CD57+	N/A	**0.0067**	0.473	0.0892
7	CD4/8mixed TD	CD4+/-CD8+/-CD57+++	N/A	**0.0166**	0.1515	0.6027
8	CD8 TD	CD8+Tbet+GrzA/B+CD57+	0.8984	**0.0107**	**0.7451**	**0.0031**
9	CD8 Tem 1	CD8+CD45ROlowTbet+GrzA+	0.3911	0.9032	0.3319	0.2464
10	CD8 Tem 2	CD8+CD45RO-Tbet-GrzA-	0.8438	**0.004**	0.3498	0.2189
11	CD8 Tex	CD8+CD45RO+CD38+PD-1++	0.6250	**0.0001**	0.2791	0.3332
12	CD8 Tnaive	CD8+CD45RA+TCF1+	N/A	**0.0001**	0.1599	0.5811

Supplementary Table 4 – Additional information for NK cell FlowSOM metaclusters.

			Metacluster frequency in PBMC vs DTC		
			Wilcoxon t-test	Spearman correlation	
**Metacluster**	**Phenotype**	**Defining Markers**	**p**	**r**	**p**
1	Naïve 1	CD56++CD16-CD57-GrzA+GrzB-	0.0676	0.2959	0.3014
2	Naïve 2	CD56dimCD16-CD57-GrzA-GrzB-CD45RA-	**0.0002**	-0.01978	0.9517
3	Naïve 3	CD56+CD16dimCD57-GrzA+GrzB+	0.2412	-0.1473	0.6158
4	Mature 1	CD56+CD16++CD8-CD57+	**0.0017**	0.178	0.5423
5	Mature 2	CD56+CD16++CD8+CD57-	**0.0029**	**0.03333**	**0.913**
6	Terminally differentiated	CD56-CD16-CD8+CD57+CD38-	0.1353	-0.1165	0.693
7	Chronically stimulated 1	CD56-CD16-CD8+CD57-Grz-Tbet-CD45RA-	0.5416	-0.1912	0.5121
8	Chronically stimulated 2	CD56-CD16-CD8-CD57-Grz-Tbet-CD45RA-HLA-DR+CD11b+	0.1531	0.002198	>0.9999
