# Supplementary material for: TIGIT can inhibit T cell activation via ligation-induced nanoclusters, independent of CD226 co-stimulation
Source: Nat Commun. 2023 Aug 18;14:5016. doi: 10.1038/s41467-023-40755-3 (PMC10439114; doi:10.1038/s41467-023-40755-3)
Supplement: Supplementary file 3 — Description of Additional Supplementary Files [file 41467_2023_40755_MOESM3_ESM.pdf]

**Description of Additional Supplementary Files**

File Name: Supplementary Video 1
Description: **3D TauSTED imaging of a Jurkat-TIGIT-SNAP-Raji-CD155 conjugation.** 2 colour stacks showing TIGIT (labelled with the SNAP label TMR-STAR; magenta) and CD155 (labelled by a V5/Secondary-AF514 immunostain; green), showing the 3-dimensional organisation of the two molecules on the cells after conjugation for 10 mins. A merged stack is also provided.

File Name: Supplementary Video 2
Description: **Live TIRF imaging of TIGIT at IS upon interaction with PLBs.** Videos showing TIGIT-SNAP, labelled with a fluorescent dye, interacting with PLBs containing 100 mols/$\mu m^2$ ICAM-1 plus either CD111 or CD155 (400 mols/$\mu m^2$; as indicated) and imaged every 3 seconds by TIRF microscopy. Scale bars = 5 µm and times are displayed as mm:ss.
