# Supplementary material for: TIGIT can inhibit T cell activation via ligation-induced nanoclusters, independent of CD226 co-stimulation
Source: Nat Commun. 2023 Aug 18;14:5016. doi: 10.1038/s41467-023-40755-3 (PMC10439114; doi:10.1038/s41467-023-40755-3)
Supplement: Supplementary file 6 — Reporting Summary [file 41467_2023_40755_MOESM6_ESM.pdf]

nature portfolio

Corresponding author(s): Daniel M. Davis

Last updated by author(s): Jul 5, 2023

Reporting Summary

Nature Portfolio wishes to improve the reproducibility of the work that we publish. This form provides structure for consistency and transparency in reporting. For further information on Nature Portfolio policies, see our Editorial Policies and the Editorial Policy Checklist.

Statistics

For all statistical analyses, confirm that the following items are present in the figure legend, table legend, main text, or Methods section.

n/a	Confirmed	
☐	☒	The exact sample size (*n*) for each experimental group/condition, given as a discrete number and unit of measurement
☐	☒	A statement on whether measurements were taken from distinct samples or whether the same sample was measured repeatedly
☐	☒	The statistical test(s) used AND whether they are one- or two-sided<br>*Only common tests should be described solely by name; describe more complex techniques in the Methods section.*
☒	☐	A description of all covariates tested
☐	☒	A description of any assumptions or corrections, such as tests of normality and adjustment for multiple comparisons
☐	☒	A full description of the statistical parameters including central tendency (e.g. means) or other basic estimates (e.g. regression coefficient) AND variation (e.g. standard deviation) or associated estimates of uncertainty (e.g. confidence intervals)
☐	☒	For null hypothesis testing, the test statistic (e.g. *F*, *t*, *r*) with confidence intervals, effect sizes, degrees of freedom and *P* value noted<br>*Give P values as exact values whenever suitable.*
☒	☐	For Bayesian analysis, information on the choice of priors and Markov chain Monte Carlo settings
☒	☐	For hierarchical and complex designs, identification of the appropriate level for tests and full reporting of outcomes
☐	☒	Estimates of effect sizes (e.g. Cohen's *d*, Pearson's *r*), indicating how they were calculated

Our web collection on statistics for biologists contains articles on many of the points above.

Software and code

Policy information about availability of computer code

Data collection	The following commercial and open source software were used for data collection in this study: Fluidigm CyTOF software (Version 6.7.1014), LASAF (v3.3; Leica), FACSDiva (v9.0.1; BD). Fiji (v1.53s), NIS-Elements AR (5.21.03; Build 1489; Nikon), Image Lab (v6.0.0.25; Bio-Rad) and Tecan i-control (v1.9.17.0).
Data analysis	The following commercial and open source software were used for data analysis in this study: FlowJo (v10.7.1 for CyTOF; 10.4.2 for all other flow cytometry data), FlowSOM (v3.0.18), UMAP (v3.1), Fluidigm CyTOF software (Version 6.7.1014), Fiji (v1.53s), ImageJ (v1.51p), R (v3.5.3), Matlab (R2018a; Mathworks), LAS X (v3.5.7; Leica), Graphpad Prism (v8.4.2), Microsoft Excel, Coloc-Tesseler (v1.0; see citation) and ThunderSTORM (dev-2014-07-16-b1; see citation). Cluster analysis of localisation microscopy data was performed using custom algorithms that are freely available at GitHub (https://github.com/quokka79/ClusterFields).

For manuscripts utilizing custom algorithms or software that are central to the research but not yet described in published literature, software must be made available to editors and reviewers. We strongly encourage code deposition in a community repository (e.g. GitHub). See the Nature Portfolio guidelines for submitting code & software for further information.

Data

Policy information about availability of data

All manuscripts must include a data availability statement. This statement should provide the following information, where applicable:

- Accession codes, unique identifiers, or web links for publicly available datasets
- A description of any restrictions on data availability
- For clinical datasets or third party data, please ensure that the statement adheres to our policy

Data supporting the findings of this study are included within the Article and its supplementary figures, tables. Videos analysed for Fig. 3f and generated for a more in-depth visualisation of Supplementary Figure 9 are included as Supplementary Videos 1–2. Uncropped immunoblots used in the manuscript are provided in the Supplementary Information. Source data are provided with this paper. The authors declare that all data can be made available upon request. Cell lines and plasmids generated for use in this study can also be made available upon request.

Human research participants

Policy information about studies involving human research participants and Sex and Gender in Research.

Reporting on sex and gender	Primary T cells were isolated from the peripheral blood of healthy blood donors purchased from the NHS blood and transplant service (NHSBT) after seeking the appropriate ethics approval. The sex of each donor was not recorded for the purposes of use in this study and both male and female donors were used.<br><br>The sex of the patients where matched tumour and peripheral blood samples originated have been provided in Supplementary Table 1. Sex was not used as a determinant for study design.
Population characteristics	No data of this nature was collected for peripheral blood donors.<br><br>For patients with matched tumour and blood samples, average age, race, current treatments, clinical stage of the tumour, smoking and alcohol use history have all been provided in Supplementary Table 1.
Recruitment	NHSBT managed and recruited blood donors that we used to isolate primary T cells, according to their screening procedures.<br><br>For matched patient tumor and peripheral blood samples, samples were acquired retrospectively from the commercial vendor Discovery Life Sciences. Samples were acquired at the time of primary tumor surgical resect.
Ethics oversight	Peripheral blood was acquired from the NHSBT under ethics license REC 05/Q0401/108 (University of Manchester) as approved by the West Midlands - Black Country Research Ethics Committee.<br><br>Tumor samples and associated reference peripheral blood samples were acquired from the commercial vendor Discovery Life Sciences under Task Order DISLS06 as approved by GSK HBSM Due Diligence.

Note that full information on the approval of the study protocol must also be provided in the manuscript.

Field-specific reporting

Please select the one below that is the best fit for your research. If you are not sure, read the appropriate sections before making your selection.

☒ Life sciences ☐ Behavioural & social sciences ☐ Ecological, evolutionary & environmental sciences

For a reference copy of the document with all sections, see nature.com/documents/nr-reporting-summary-flat.pdf

Life sciences study design

All studies must disclose on these points even when the disclosure is negative.

Sample size	No statistical methods were used to predetermine the sample size. Sample size required a minimum of 3 independent repeats (for cell lines) and 3 individual donors (for primary cells), in line with our previous experience with similar experimentation (https://doi.org/10.1126/scisignal.aaw9252; https://doi.org/10.1073/pnas.2010274117; https://doi.org/10.1002/jev2.12215). The sample size is provided in the appropriate figure legends.
Data exclusions	As stated in Supplementary Figure 1 legend, "any portion of [CyTOF] data where signals were variable across time were removed manually". No other data was excluded from analysis.
Replication	All experiments were carried out independently at least 3 times. All work with primary human cells were performed with 3 independent blood donors, and carried out independently. Additionally, we endeavoured to replicate observations in cell lines with synthetic protein constructs

in primary cells with endogenously expressed forms. All replicates were included, and all information regarding replication is provided in the relevant figure legends.

Randomization

Randomisation was not relevent to the in vitro experiments performed within the manuscript. All data was analysed uniformly often using unbiased computational approaches.

Blinding

No blinding was performed on samples within this manuscript. Experiments required a priori knowledge of the cells or treatment groups, and were most often performed by a single person, making blinding difficult. All results, however, were assessed uniformly and in an unbiased manner, with no data exclusions apart from that listed above.

Reporting for specific materials, systems and methods

We require information from authors about some types of materials, experimental systems and methods used in many studies. Here, indicate whether each material, system or method listed is relevant to your study. If you are not sure if a list item applies to your research, read the appropriate section before selecting a response.

Materials & experimental systems

n/a	Involved in the study
☐	☒ Antibodies
☐	☒ Eukaryotic cell lines
☒	☐ Palaeontology and archaeology
☒	☐ Animals and other organisms
☒	☐ Clinical data
☒	☐ Dual use research of concern

Methods

n/a	Involved in the study
☒	☐ ChIP-seq
☐	☒ Flow cytometry
☒	☐ MRI-based neuroimaging

Antibodies

Antibodies used

All antibodies used in the study are described in detail within the manuscript, with the manufacturer name, clone number, application and concentration stated.

The stimulatory TCR antibody (OKT3) was first monobiotinylated, as described in the manuscript, to be used in planar lipid bilayers. OKT3 was produced in house.

Antagonistic TIGIT antibody (Clone VSIG9.01; produced by the Center for proteomics, Faculty of Medicine, University of Rijeka, Used at 5 μg/mL in imaging assays and 22.5 μg/mL in the 6-hour cytokine secretion assays)

For Flow cytometry (all antibodies used at the supplier's recommended dilutions):
αTIGIT (Clones MBSA43 [#16-9500-82, Thermo Fisher Scientific] and A15153G [372702, BioLegend]), αCD111 (Clone R1.302; 340404; BioLegend), αCD155 (Clone SKII.4; 337622; BioLegend) and αDNAM-1 (Clone DX11; #MA5-28150; Invitrogen). For isotype controls, the clone MOPC-21 (400166; BioLegend) was used for mouse IgG1 κ antibodies and the clone MOPC-173 (400264; BioLegend) for mouse IgG2a κ antibodies

For immunofluorescence:
αTIGIT (MBSA43; 2.5 μg/mL), GFP-Booster nanobodies (Atto488-, Alexa Fluor 488- and Alexa Fluor 647-conjugated; gba488, gb2AF488 and gb2AF647; ChromoTek; 1 μg/mL), αDNAM-1 (Clone DX11; 2.5 μg/mL), αCD96 (Clone NK92.39; 5 μg/mL), αV5 (Rabbit polyclonal; NB600-381; Novus Biologicals; 1 μg/mL), αCD19 (Clone HIB19; 302250; BioLegend; 2 μg/mL), αCD4 (Clone MT310; sc-19641; Santa Cruz Biotechnology; 2 μg/mL) and αCD8 (Clone RPA-T8; 301062; 2.5 μg/mL).

For Western Blotting:
TIGIT (E5Y1W; 1:2000; Cell Signaling Technology), Beta-Actin-HRP (GTX109639; 1:10000; GeneTex), CD3ζ (6B10.2; 1:1000; sc-1239; Santa Cruz Biotechnology), pCD3ζ (Y142; 1:1000; K25-407.69; #558402; BD Biosciences), Zap70 (D1C10E; 1:1000; #3165; Cell Signaling Technology), pZap70 (Y319; 1:1000; #2701; Cell Signaling Technology), LAT (E3U6J: 1:1000; #45533; Cell Signaling Technology), pLAT (Y220; 1:1000; #3584; Cell Signaling Technology), ERK1/2 (#9102; 1:1000; Cell Signaling Technology), pERK1/2 (E10; 1:2000; #9106; Cell Signaling Technology), AKT (C67E7; 1:1000; #5373; Cell Signaling Technology), pAKT (S473; E4U3U; 1:1000; #23430; Cell Signaling Technology), IκBα (L35A5; 1:1000; #4814; Cell Signaling Technology), pIκBα (S32; 14D4; 1:1000; #2859; Cell Signaling Technology), αRabbit IgG-HRP (#7074; Cell Signaling Technology) and αMouse IgG-HRP (#7076; Cell Signaling Technology) antibodies were used.

For mass cytometry, 21 purified antibodies were custom conjugated to metals in house using Fluidigm metal conjugation kits and the remainder were commercially purchased (see Table S2). All mass cytometry antibodies were validated and titrated as described in the box below.

Metal, Tag, Target, Clone, Conjugation (purified mAb vendor), Dilution*
89Y CD45 HI30 Fluidigm 1:100
106Cd CD45 HI30 In house (BioLegend) 1:100
110Cd CD45 HI30 In house (BioLegend) 1:200
111Cd CD3 UCHT1 In house (BioLegend) 1:50
112Cd HLA-DR L243 In house (BioLegend) 1:200
113Cd CD4 RPA-T4 In house (BioLegend) 1:100

	114Cd CD11b ICRF44 In house (BioLegend) 1:100<br>116Cd CD8a RPA-T8 In house (BioLegend) 1:100<br>141Pr CD28 CD28.2 In house (BioLegend) 1:50<br>142Nd CD19 HIB19 Fluidigm 1:200<br>143Nd CD127 A019D5 Fluidigm 1:50<br>n/a CX3CR1-FITC 2A9-1 n/a (BioLegend) 1:50<br>144Nd FITC FIT22 Fluidigm 1:50<br>145Nd TCF1 7F11A10 In house (BioLegend) 1:100<br>146Nd CD11c 3.9 Fluidigm 1:100<br>147Sm Granzyme A CB9 In house (BioLegend) 1:100<br>148Nd PD-1 EH12.2H7 In house (BioLegend) 1:100<br>149Sm CD45RO UCHL1 Fluidigm 1:100<br>151Eu CD14 M5E2 Fluidigm 1:100<br>152Sm FoxP3 PCH101 In house (eBioscience) 1:200<br>153Eu CD206 15-2 In house (BioLegend) 1:100<br>154Sm TIGIT MBSA43 Fluidigm 1:50<br>155Gd CD56 B159 Fluidigm 1:50<br>156Gd IFNg 4S.B4 In house (eBioscience) 1:100<br>158Gd CD96 In house In house 1:50<br>159Tb PD-L1 29E.2A3 Fluidigm 1:50<br>160Gd Tbet 4B10 Fluidigm 1:200<br>161Dy CD107a H4A3 In house (BioLegend) 1:1600<br>163Dy CCR8 L263G8 In house (BioLegend) 1:25<br>164Dy CD15 W6D3 Fluidigm 1:100<br>165Ho CD101 BB27 Fluidigm 1:100<br>166Er NKG2D ON72 Fluidigm 1:50<br>167Er CD112 TX31 In house (BioLegend) 1:100<br>168Er Ki-67 B56 Fluidigm 1:1600<br>169Tm CD25 2A3 Fluidigm 1:400<br>170Er CD45RA HI100 Fluidigm 1:800<br>171Yb CD226 DX11 Fluidigm 1:50<br>172Yb CD38 HIT2 Fluidigm 1:100<br>173Yb Granzyme B GB11 Fluidigm 1:800<br>174Yb CD57 HNK-1 In house (BioLegend) 1:3200<br>175Lu CD155 SKII.4 In house (BioLegend) 1:100<br>176Yb ICOS C398.4A In house (BioLegend) 1:200<br>Ir191/193 DNA n/a Fluidigm 1:2000<br>198Pt Cisplatin n/a Fluidigm 1:4000<br>209Bi CD16 3G8 Fluidigm 1:50<br>*Note in house conjugated antibodies are at 0.5 mg/mL prior to labelling.
Validation	Antibodies used for for TIGIT, CD96, GFP, V5, CD111 and CD155 were all validated using cell lines lacking endogenous expression where we exogenously expressed constructs, providing positive and negative control samples. For IF and WB, all other antibodies were purchased based on their use in published research. All antibodies were purchased from well established companies known for antibody manufacturing and sale and were validated by the manufacturer for use in their respective assays and against their listed, cognate antigen. All statements and citations of validation can be found on the product website from each company. Product numbers have been provided.<br><br>For mass cytometry, all markers were validated and titrated in house using a variety of cell types (PBMC, DTC, and cell lines) and culture conditions. Metal minus one (MMO) and metal minus many (MMM) panels were used during panel optimization to ensure there was no signal overlap among channels.

Eukaryotic cell lines

Policy information about cell lines and Sex and Gender in Research

Cell line source(s)	All cell lines presented in the manuscript were originally purchased from the ATCC.
Authentication	No authentication of cell lines was performed.
Mycoplasma contamination	Cell lines were tested regularly for mycoplasma contamination and always tested negative.
Commonly misidentified lines<br>(See ICLAC register)	No commonly misidentified cell lines were used.

Flow Cytometry

Plots

Confirm that:

☒ The axis labels state the marker and fluorochrome used (e.g. CD4-FITC).

☒ The axis scales are clearly visible. Include numbers along axes only for bottom left plot of group (a 'group' is an analysis of identical markers).

☒ All plots are contour plots with outliers or pseudocolor plots.

☒ A numerical value for number of cells or percentage (with statistics) is provided.

Methodology

Sample preparation	For flow cytometry: To evaluate the abundance of cell surface proteins, living cells were washed in DPBS, and stained with a LIVE/DEAD dye (Zombie, 1:1000 dilution; BioLegend) for 20 min at 4°C. After subsequent DPBS washing, cells were then fixed with 4% paraformaldehyde at 37°C for 15 mins. Fixed cells were then washed in DPBS before being blocked in DPBS containing 3% Bovine serum Albumin and 1% Human serum for 20 mins at RT. Cells were then stained in blocking buffer with the indicated primary antibodies for 30 mins at RT. Where secondary antibodies were needed, unbound primary antibodies were washed off with 3 DPBS washes followed by another 30 mins incubation of the secondary antibodies in blocking buffer. Cytoplasmic stains were performed, as above, but with the inclusion of a 5 mins 0.1% Triton-X100 permeabilisation step following fixation.<br>For mass cytometry: Frozen single cell suspensions of blood and tumour cells were thawed in a 37°C water bath, slowly added into pre-warmed AIM-V media (Gibco), washed and resuspended in 1mL AIM-V prior to filtering through a FlowMi® 70uM Cell Strainer (Sigma Aldrich). Cells were plated in a 96-well round-bottom polystyrene plate (Costar) at 2-4x106 cells per well. Cells were first stained with 0.25µM Cisplatin-198Pt in Maxpar® PBS (Fluidigm) for 5 mins, washed, and resuspended in Maxpar® Cell Staining Buffer (Fluidigm) containing Human TruStain FcXTM (BioLegend) for 10 mins. Next the cells were stained with a unique CD45 antibody and the primary surface antibody cocktail for 20 mins. The final surface stain was performed with the secondary anti-FITC antibody under the same conditions. Cells were then fixed and permeabilized using eBioscience™ Foxp3/Transcription Factor Staining Buffer Set (Thermo Fisher Scientific) for 45 mins at 4°C. After permeabilization, cells were incubated for 10 mins with Human TruStain FcXTM prior to staining with the intracellular antibody cocktail for 30 mins. Finally, cells were resuspended in Cell-ID™ Intercalator-Ir (Fluidigm) in Maxpar® Fix/Perm Buffer (Fluidigm). The 96-well plates were then sealed and stored at 4°C for 24-72 hours prior to acquisition on a CyTOF2 mass cytometer (Fluidigm) equipped with a Super Sampler (Victorian Airship and Scientific Apparatus, LLC).
Instrument	Flow cytometric analysis was carried out on either a BD Fortessa X20 or a BD FACSymphony (Becton Dickinson).<br><br>Mass cytometry: samples were acquired on a CyTOF2 mass cytometer (Fluidigm) equipped with a Super Sampler (Victorian Airship and Scientific Apparatus, LLC).
Software	Flow cytometry: Acquisition was performed with BD FACSDiva (v9.0.1) and all analysis was carried out with FlowJo (v10.4.2; FlowJo).<br>Mass cytometry: data was randomized and normalized using Fluidigm CyTOF software (Version 6.7.1014) and all downstream analysis was carried out with FlowJo (v10.7.1; FlowJo).
Cell population abundance	Cell subsets isolated by magnetic bead separation were periodically assessed for purity with the appropriate surface markers (CD4 and CD8), to ensure we achieved the purities specified by the manufacturer.
Gating strategy	Flow cytometry: Cells are gated with FSC/SSC to remove cell debris, followed by gating single cells based on FSC-A/FSC-W and then gating of the LIVE/DEAD marker.<br>Mass Cytometry: First, live single cells were identified based on exclusion of beads, exclusion of cisplatin, incorporation of DNA intercalator, and event length. Unbiased high-dimensional analysis was used to determine the phenotype of T cells and NK cells in our sample set (data not shown), which then guided biaxial gating strategies to identify CD4+ Tregs, CD4+ T cells (non-Tregs), CD8+ T cells, and NK cells. Based on the data, two gating modifications were made to accommodate cells present in the tumour samples that expressed very high levels of HLA-DR in tumour cells and the gating strategies are shown in extended data figure 1.

☒ Tick this box to confirm that a figure exemplifying the gating strategy is provided in the Supplementary Information.
