# Supplementary material for: TIGIT can inhibit T cell activation via ligation-induced nanoclusters, independent of CD226 co-stimulation
Source: Nat Commun. 2023 Aug 18;14:5016. doi: 10.1038/s41467-023-40755-3 (PMC10439114; doi:10.1038/s41467-023-40755-3)

Uncropped immunoblots

Figure 7g

20 μm Phos-tag SDS-PAGE
αTIGIT

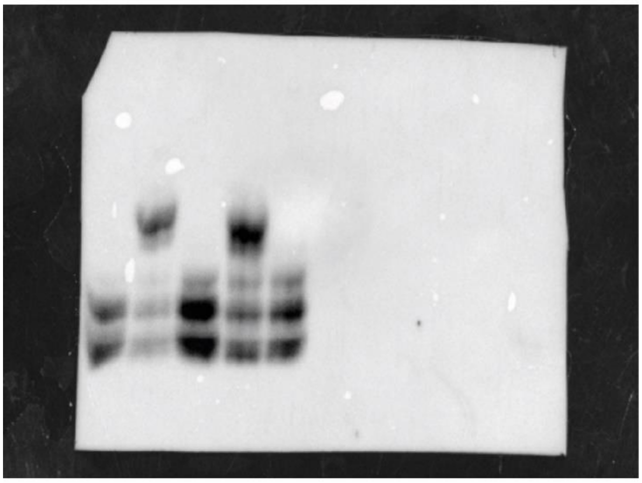

Standard SDS-PAGE
αTIGIT

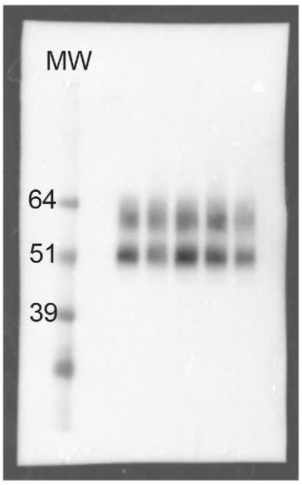

Supplementary Figure 15b

αTIGIT

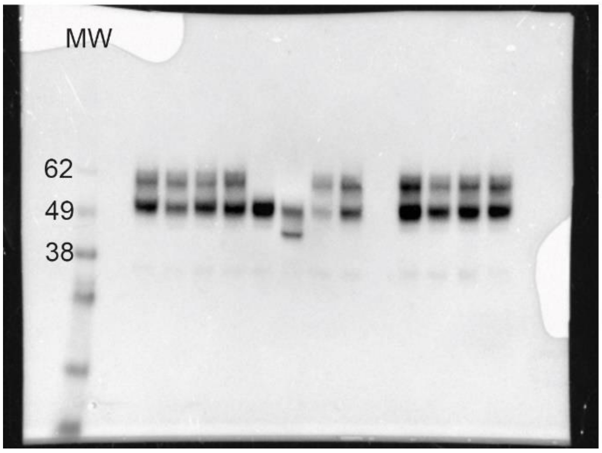

β-Actin

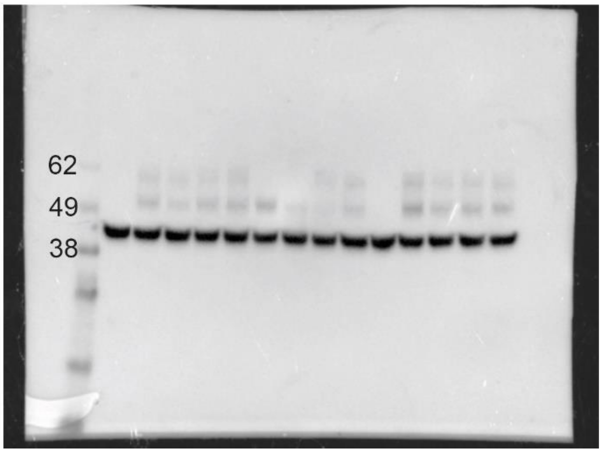

Supplementary Figure 16

pCD3ζ (Y142) & pZap70 (Y319)

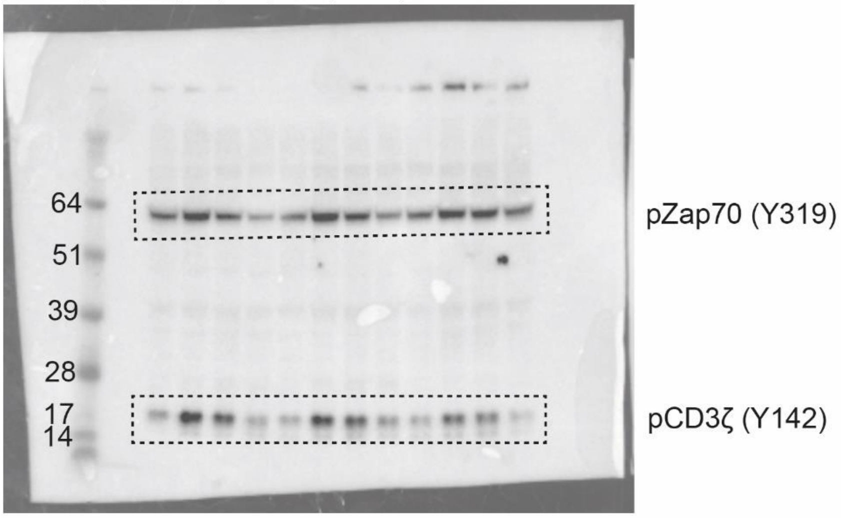

pLAT (Y220)

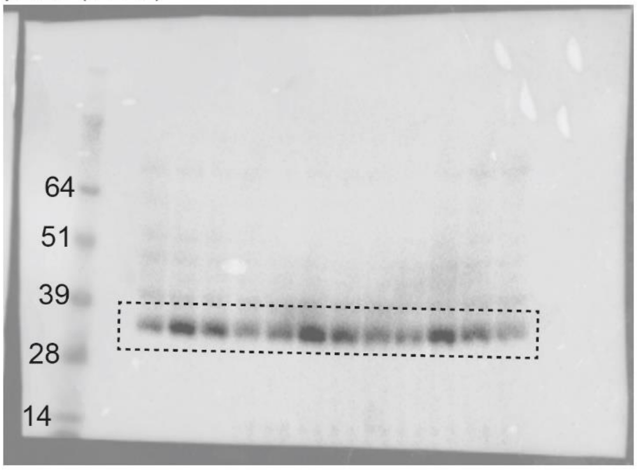

pIκBα (S32)

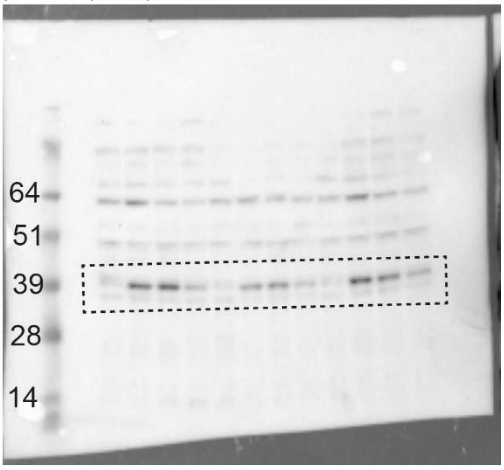

pERK1/ERK2 (T202/Y204) & pAKT (S473)

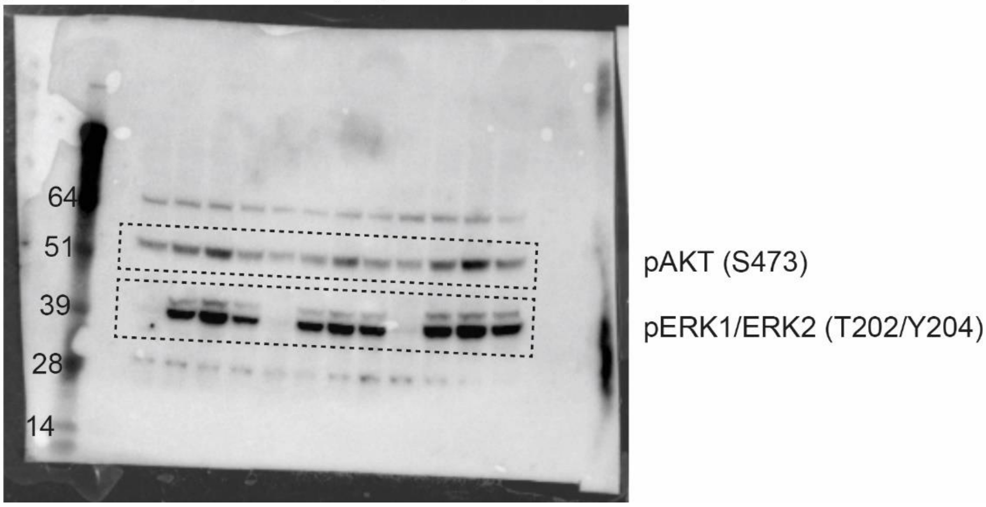

Supplementary Figure 16 (continued)

CD3ζ

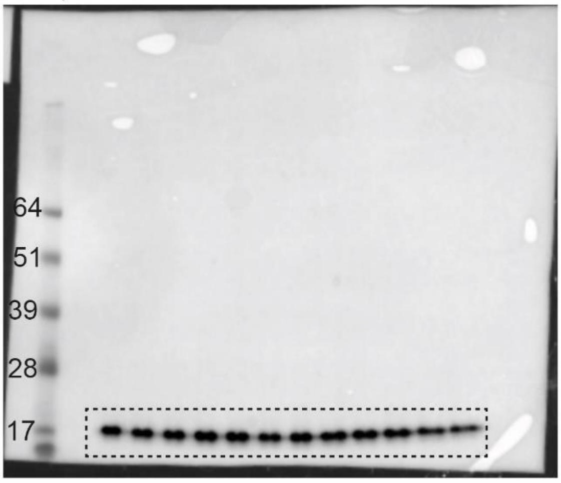

AKT

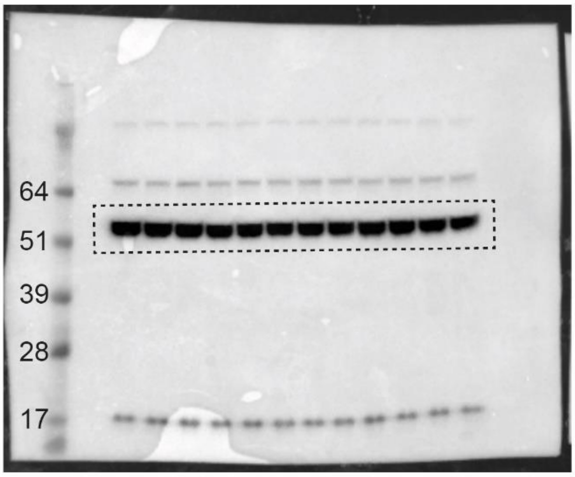

Zap70

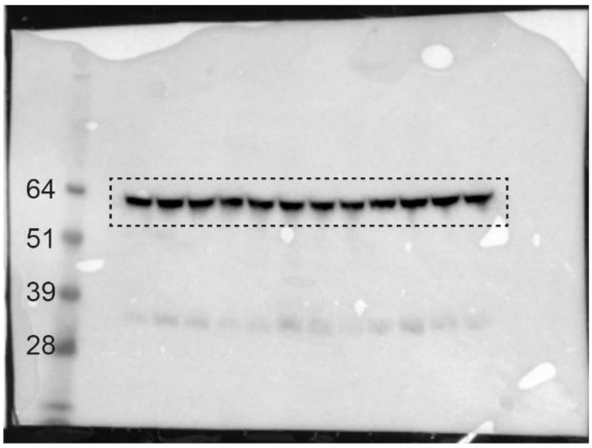

LAT

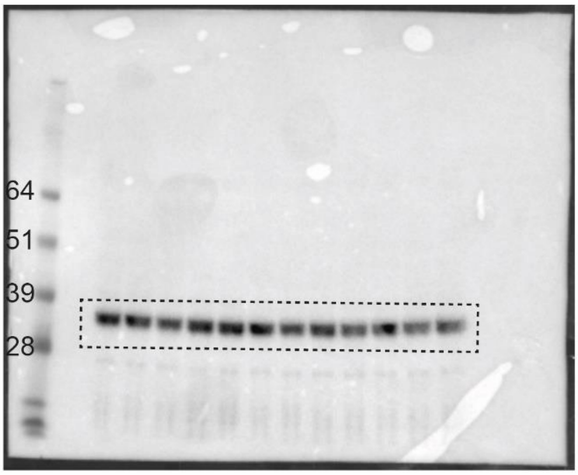

ERK1/ERK2

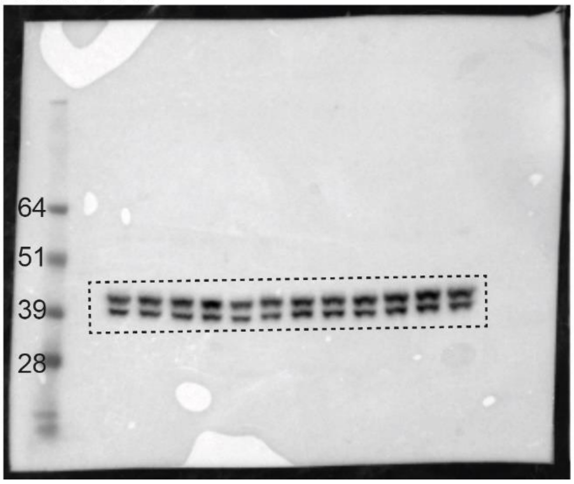

IκBα

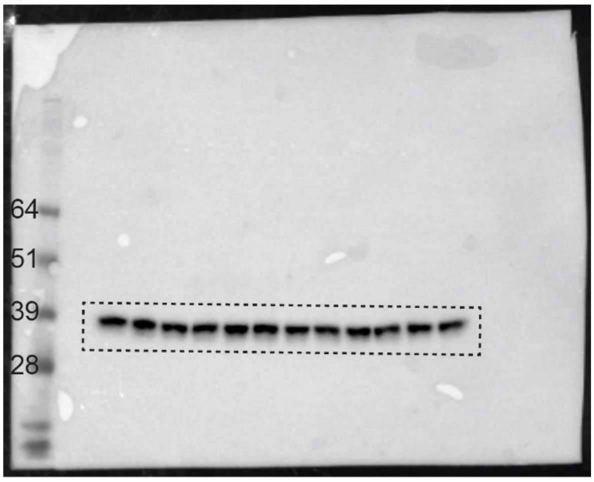

Supplementary Figure 16 (continued)

TIGIT

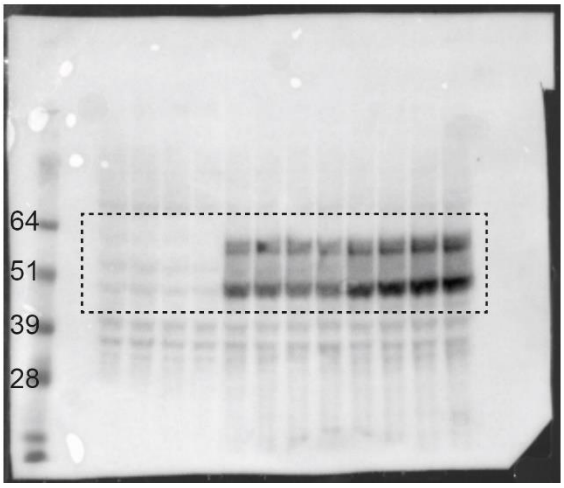

β-Actin

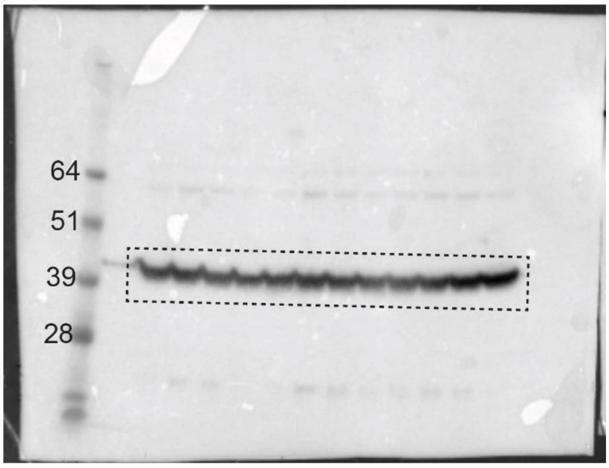

Supplement: Supplementary file 7 — Source Data [file 41467_2023_40755_MOESM7_ESM.zip › Source Data File 2 - Uncropped Immunoblots.pdf]
